# Incorporating structural similarity into a scoring function to enhance the prediction of binding affinities

**DOI:** 10.1186/s13321-021-00493-4

**Published:** 2021-02-15

**Authors:** Beihong Ji, Xibing He, Yuzhao Zhang, Jingchen Zhai, Viet Hoang Man, Shuhan Liu, Junmei Wang

**Affiliations:** Department of Pharmaceutical Sciences and Computational Chemical Genomics Screening Center, School of Pharmacy, University of Pittsburgh, Pittsburgh, PA 15261 USA

**Keywords:** Scoring function, Virtual screening, Docking, Compound similarity, Fingerprint, Protein‐ligand binding, Computer‐aided drug design

## Abstract

In this study, we developed a novel algorithm to improve the screening performance of an arbitrary docking scoring function by recalibrating the docking score of a query compound based on its structure similarity with a set of training compounds, while the extra computational cost is neglectable. Two popular docking methods, Glide and AutoDock Vina were adopted as the original scoring functions to be processed with our new algorithm and similar improvement performance was achieved. Predicted binding affinities were compared against experimental data from ChEMBL and DUD-E databases. 11 representative drug receptors from diverse drug target categories were applied to evaluate the hybrid scoring function. The effects of four different fingerprints (FP2, FP3, FP4, and MACCS) and the four different compound similarity effect (CSE) functions were explored. Encouragingly, the screening performance was significantly improved for all 11 drug targets especially when CSE = S^4^ (S is the Tanimoto structural similarity) and FP2 fingerprint were applied. The average predictive index (PI) values increased from 0.34 to 0.66 and 0.39 to 0.71 for the Glide and AutoDock vina scoring functions, respectively. To evaluate the performance of the calibration algorithm in drug lead identification, we also imposed an upper limit on the structural similarity to mimic the real scenario of screening diverse libraries for which query ligands are general-purpose screening compounds and they are not necessarily structurally similar to reference ligands. Encouragingly, we found our hybrid scoring function still outperformed the original docking scoring function. The hybrid scoring function was further evaluated using external datasets for two systems and we found the PI values increased from 0.24 to 0.46 and 0.14 to 0.42 for A2AR and CFX systems, respectively. In a conclusion, our calibration algorithm can significantly improve the virtual screening performance in both drug lead optimization and identification phases with neglectable computational cost.
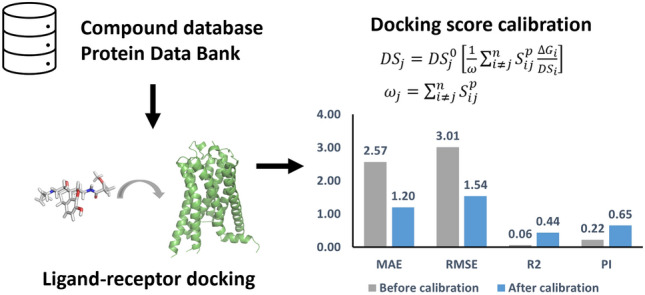

## Introduction

In order to save time and cost in drug discovery projects, various in silico approaches have been developed and applied to reduce the number of compounds which are to be experimentally synthesized and tested. Among these computer-aided drug design/discovery (CADD) methods, virtual screenings of large chemical databases for potential bioactive molecules are usually conducted at the very beginning stage to rapidly narrow down the candidates from millions of compounds to manageable numbers (thousands or hundreds). Depending on whether the 3D structural information of the target receptor is available and utilized, virtual screening (VS) methods can be broadly classified into structure-based (SBVS) and ligand-based (LBVS) [1]. Docking & scoring is a typical SBVS method, which predicts whether a ligand can favorably interact with a receptor (protein or nucleic acid) at its binding site, and if yes, the binding mode and binding affinity measured by the docking scoring function are determined. [1, 2]. Similarity search is a typical LBVS method, which predicts activity of query compounds depending on their similarities/dissimilarities to known reference ligands by utilizing numerical similarity descriptors (fingerprints) [3]. Both docking and similarity methods have been successfully carried out independently or hierarchically to screen out confidently inactive compounds for specific receptors of interest. Compared to docking, similarity search is even faster, and therefore it is suitable to filter super large database before docking takes place. However, both similarity search and docking suffer from poor accuracy to rank potentially active ligands and prioritize top candidates to be suggested for experiments. Similarity search is based on the Similarity Property Principle (SPP) [4], i.e., structurally similar molecules are likely to possess similar biological properties and activities. However, this hypothesis is not always true. Sometimes small chemical differences may arise highly different activity (‘activity cliffs’) [5]. Accuracy of docking methods is limited due to lack of modelling structural flexibility of target receptors, effects of solvation and entropy changes, etc. These limitations of docking & scoring methods may be overcome by more accurate methodologies, such as end-point methods (MM-PBSA, MM-GBSA, LIE, etc.) [6, 7], or rigorous alchemical free energy methods (FEP, TI, etc.) [8, 9], with the price of much higher computational cost and much longer time. But we cannot help wondering: is there a way to improve the accuracy of docking & scoring methods without much more extra computational cost? With the advances of high-throughput screening technique, more and more compounds were measured against a drug target. ChEMBL [10] is a curated database which collects binding affinities of bioactive molecules for a drug target. How can we utilize the information on the known structures and activities to improve screening performance? Secondly, can we combine docking & scoring methods with the extremely fast methods of similarity calculations to improve the accuracy of binding affinity estimation? If so, how can we incorporate the two types of scores into one hybrid scoring function? In this work, we attempted to develop a novel algorithm to make a good use of those valuable information on known bioactive compounds.

Docking programs utilize scoring functions to estimate the binding affinities. Currently, scoring functions can be generally classified into following four categories based on how protein-ligand energy is predicted: (1) force-field-based, (2) empirical, (3) knowledge-based, (4) descriptor-based [11]. Although the underlying mechanisms of four categories are different, all of those developed scoring functions are trying to pursue promising results in the protein-ligand binding prediction. Considering the progress and achievements that have already been made by the existing scoring functions during the past few decades, we believe it will be more valuable to make some improvements based on current developed scoring functions. Therefore, in this work, instead of developing a completely novel scoring function for the binding affinity prediction, we introduced a more practical and universal approach that can improve the scoring power for an arbitrary docking scoring function. Our new scoring algorithm is suitable to the following scenario: the binding affinities against a specific receptor for a certain number of compounds (hereafter called reference compounds or reference ligands) have been experimentally measured, but many more compounds (hereafter called query compounds) need to be estimated in silico. Such a scenario is frequently encountered in real drug design projects. Two components were incorporated to compose our new algorithm: (1) the structural difference between the query compounds and reference ligands; (2) the deviation of the docking scores of those reference ligands and their experimental binding affinities. Both the above components can serve as weights to calibrate the original docking scores of ligands.

## Materials and methods

### Work outline

**Fig. 1:**
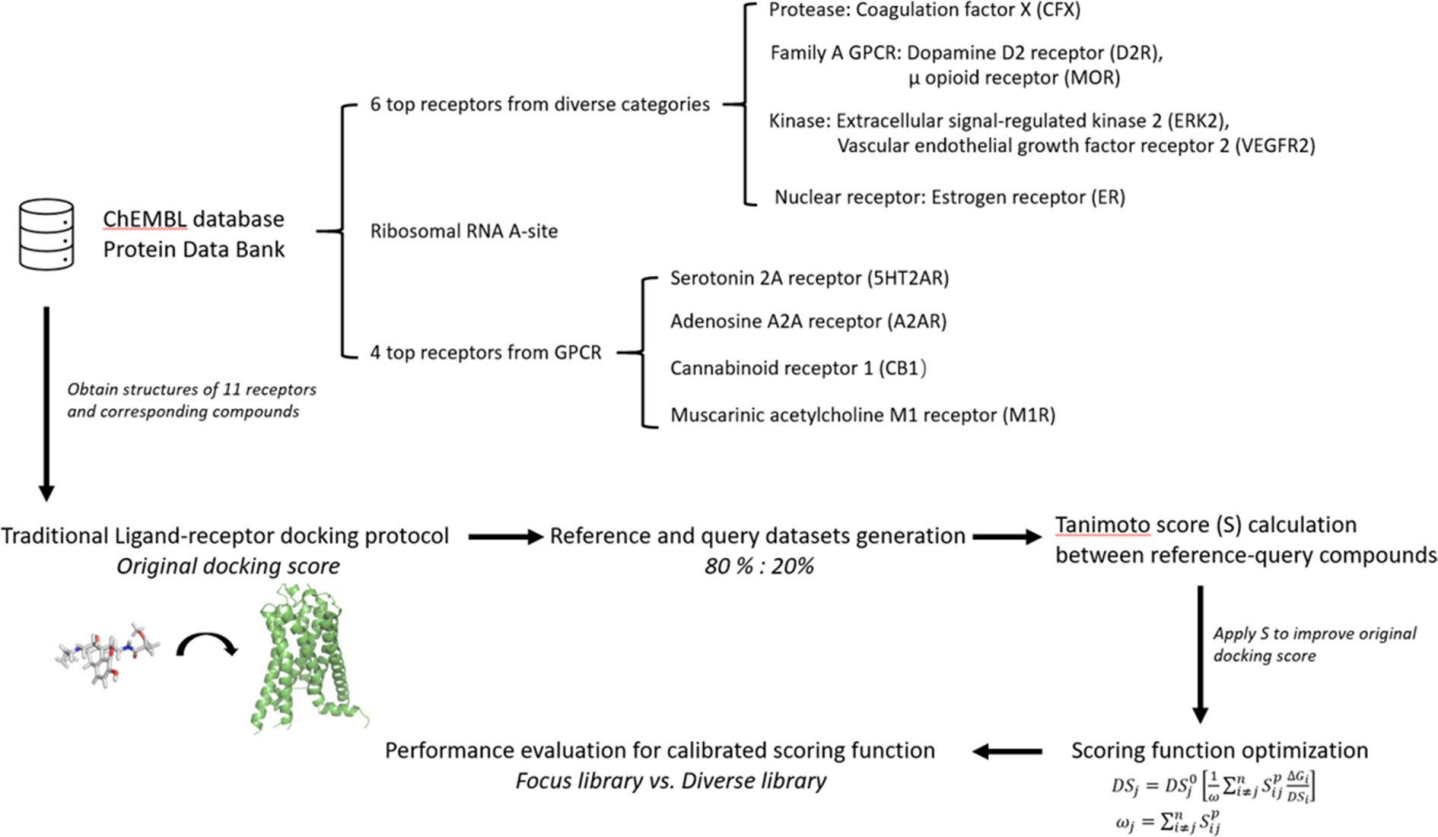
The flowchart of the calibration algorithm

The outline of our work is shown in the flow chart of Fig. 1. To evaluate the feasibility and performance of our algorithm, we collected the X-ray crystal structures of 11 receptors of various categories from the Protein Data Bank [12] (https://www.rcsb.org) and their available ligand data from the ChEMBL database [13, 14] (https://www.ebi.ac.uk/chembl/). Collected compounds for each receptor were divided into reference set (reference ligands) and validation set (query compounds). See Table 1 and Additional file 1. Compounds were docked to their corresponding targets with a state-of-art docking program Glide, which was selected as the basic scoring function for further improvement [15, 16]. The Tanimoto Coefficient Tc [3] was calculated by utilizing the Open Babel program version 2.3.1 (http://openbabel.org) [17]. Four popular 2D fingerprints (FP2, FP3, FP4, MACCS) [17] available in the Open Babel package were adopted and compared in this study. The proposed hybrid scoring function was applied to calibrate the Glide docking scores. The scoring and ranking power of the hybrid scoring function as well as the original Glide docking scoring function was measured by root-mean-square-error (RMSE), mean-absolute-error (MAE), Pearson’s correlation coefficient (R^2^), and the predictive index (PI) between the docking scores and the experimental binding affinities [18–20]. In addition, we evaluated the screening power of the hybrid scoring function by using enrichment factor (EF) and the area under the curve (AUC) of receiver operating characteristic (ROC) curve [21]. We also explored the choice of fingerprint type and CSE function form to optimize the screening performance. More details about the preparation of receptors and ligand datasets, docking software and procedures, and methods of evaluations are described in the following sessions.

**Table 1:** The information data of compounds for 11 receptors

Receptor	Total Compounds	Activities (K_i_/K_d_)	Reference set	Validation set
CFX	7084	4521	581	144
D2R	9897	10,473	635	161
ERK2	19,729	1767	690	171
ER	6561	1452	191	51
MOR	7939	5796	362	110
VEGFR2	12,497	1521	509	121
5HT2AR	6524	5130	619	150
A2AR	9015	7172	723	166
CB1	8907	5544	529	117
M1R	4858	2196	469	108
rRNA	79	77	57	14

### Preparation of receptor datasets

To test the developed algorithm in this study, 11 receptors were selected according to the experimental records in ChEMBL database. The receptors can be divided into three classes: (1) 6 top receptors from diverse categories; (2) 4 top receptors from GPCR family; (3) one RNA receptor. Coagulation factor X (CFX), dopamine D2 receptor (D2R), µ opioid receptor (MOR), Extracellular signal-regulated kinase 2 (ERK2), vascular endothelial growth factor receptor 2 (VEGFR2) and estrogen receptor (ER) are in class (1). Among them, CFX is a member of Protease. D2R and MOR are from family A GPCR. EKR2 and VEGFR2 are members of the kinase family, while ER is the nuclear receptor. In class (2), there are serotonin 2A receptor (5HT2AR), adenosine A2A receptor (A2AR), cannabinoid receptor 1 (CB1) and muscarinic acetylcholine M1 receptor (M1R). Finally, in class (3), the ribosomal RNA (rRNA) A-site was selected as the receptor. All the receptor categories were selected according to the rank of member amounts in their category families, while the receptors themselves were selected based on the compound number recorded in binding assays. The above information was collected from ChEMBL database and is shown in Table 1. Then the X-ray crystal structures of the above 11 target receptors were retrieved from Protein Data Bank (detail information was shown in Additional file 1: Table S1). The sources of all targets are Homo sapiens.

### Preparation of ligand datasets

To better compare the binding energy of each ligand that binds to the same receptor, we collected 3D structures (SDF format) of ligands with the inhibition constant (K_i_) values recorded from binding assays in the ChEMBL database. For the ribosomal RNA receptor, because of the limited number of K_i_ activities, compounds with dissociation constant (K_d_) values were collected. It is of note that the activities of those collected compounds for each receptor were measured using the same methods. To balance the distribution of K_i_ activities for each receptor, compounds were hierarchically classified into 4 levels according to their K_i_ values: K_i_ < 10 nM, 10 nM ≤ K_i_ < 1 µM, 1 µM ≤ K_i_ < 100 µM and K_i_ ≥ 100 µM. In each level, 300 or less than 300 compounds (if the number of compounds in the level does not reach 300) were randomly collected by utilizing numpy.random.choice in Python 3.7 program [22]. For one selected compound with 2 or more K_i_ values that came from various assays, the average K_i_ was used. To evaluate the screening power of our approach, we categorized the selected compounds into the active and inactive sets by the cutoff of K_i_/K_d_ = 100 nM. This value is lower than normal threshold, 10 µM, but it can better balance the numbers of compounds in the active and inactive sets. The experimental K_i_/K_d_ for each collected compound was then converted to the experimental ligand-receptor binding energy (kcal/mol) by the Eq. 1.

1 $$\varDelta {G}_{binding}= -RTln{K}_{i \left(d\right)}$$

where $\varDelta {G}_{binding}$ is the binding energy of the ligand, R is the gas constant with a value of 8.314 J mol^−1^ K^−1^ and T is the room temperature under standard pressure with the value of 298.15 K.

To exclude compounds with very weak binding affinities and make our evaluation more reliable, compounds for 11 receptors with experimental binding energy higher than − 4 kcal/mol were removed from the selected datasets. Then we randomly separated compounds into training datasets and testing datasets by the proportion of 4:1. The number of compounds in training and testing sets for each receptor was listed in Table 1. The structures of compounds were not only stored in the mol2 format but also converted into different 2D fingerprints for the further exploration of our algorithm by Open Babel program, which is an expert chemical toolbox for the format interconversion of chemical data [23]. Four 2D fingerprints are available in OpenBabel according to its documentation (http://openbabel.org/docs/dev/Features/Fingerprints.html): (1) FP2, a path-based fingerprint stored in a 1024-bit vector; (2) FP3, s series of SMARTS queries stored in 55 bits; (3) FP4, s series of SMARTS queries stored 307 bits; (4) MACCS, a series of SMARTS patterns stored in 166 bits.

To critically evaluate the performance of our calibration algorithm, we performed extra validation test on hundreds of compounds with activities (K_i_) of A2AR and CFX from an additional database, DUD-E database [24, 25]. After excluding the compounds with binding energy higher than − 4 kcal/mol and those already included in the reference datasets, two external test datasets which have 1973 and 1599 unique compounds were compiled for the A2AR and CFX systems, respectively. All compounds from DUD-E were treated as query molecules and their docking scores were calibrated with the compounds in the corresponding ChEMBL dataset as references.

### Docking software and procedure

We docked selected compounds (include training sets, test sets and external test sets) to their corresponding receptors utilizing the Glide docking program implemented in the Schrodinger software (Maestro 11.2). Before docking, the downloaded SDF files of ligands were processed with the *LigPrep* module in Maestro. The downloaded PDB files of receptors were processed with the module of *Protein Preparation Wizard* in Maestro: removing co-crystallized solvent and ions, adding hydrogen atoms and missing site-chain atoms, energy minimization on hydrogen atoms. Then we defined the binding site based on the geometric center of the native bound ligand without taking constraint or rotatable group into consideration. The flexible docking with post-docking minimization for 11 systems was conducted by the following settings: van der Waal radius scaling factor was 0.80, the partial charge cutoff for ligands was 0.15, the intramolecular hydrogen bond formation was rewarded, the number of poses per ligand to include was 10. The top binding pose with the best docking energy score was retained and stored.

To investigate the impact of different docking scoring function on our calibration algorithm, the performance of our hybrid scoring functions was also evaluated for the AutoDock Vina docking scoring function [26]. Again, selected compounds were docked to their corresponding receptors utilizing the AutoDock Vina docking program. The receptor preparation was performed following the same protocol in Glide docking program. The binding site and space were defined based on the geometric center and the size of the native bound ligand without taking constraint or rotatable group into consideration. Considering the different docking mechanisms of two docking programs, this time, compounds with experimental binding energy higher than − 5 kcal/mol were removed from the selected datasets to exclude compounds with weak binding affinities. Then compounds were randomly separated into training datasets and testing datasets proportionally and the docking score was calibrated using our proposed calibration approach. The Glide docking scores and AutoDock Vina docking scores as well as the experimental binding affinities (converted from K_i_ values) of compounds in the reference and validation sets were listed in Additional file 2: Table S2A, Additional file 3: Table S2B.

### Algorithm for docking score calibration

The new algorithm we proposed to calibrate the docking scores from a normal docking program is described as below:

2 $${DS}_{j}={DS}_{j}^{0}\left[\frac{1}{\omega }{\sum }_{i\ne j}^{n}{S}_{ij}^{p}\frac{{\varDelta G}_{i}}{{DS}_{i}}\right]$$

3 $$\omega ={\sum }_{i\ne j}^{n}{S}_{ij}^{p}$$

where ${DS}_{j}^{0}$ and ${DS}_{j}$ are the docking score of the *j*th query compound before and after the calibration. ${S}_{ij}$ is the structural similarity between the *j*th query compound and the *i*th reference ligand. The exponent p is treated as an integer constant with its value varying from 1 to 4 in this study, for the exploration of the developed formula. We referred ${S}_{ij}^{p}$ as compound similarity effect (CSE) function for convenience of discussion. *n* is the total number of reference ligands in the reference dataset. ${DS}_{i}$ is the docking score of the *i*th reference ligand. ${\varDelta G}_{i}$ is the experimental binding energy (kcal/mol) of the *i*th compound in the reference dataset, which is converted from the experimental K_i_/K_d_ by the Eq. (1).

In this study, the structural similarity ${S}_{ij}$ between two compounds is represented by the Tanimoto Coefficient (Tc) calculated from their 2D fingerprints: [3]

4 $${T}_{c}\left(X,Y\right)=\frac{z}{\text{x}+\text{y}-\text{z}}$$

where x and y are the number of bits in the fingerprints of compounds X and Y, z is the number of bits set shared by compounds X and Y. Tc has a range between 0 and 1 and a larger value means higher structural similarity between two compounds. The Tc calculation was carried out by utilizing Open Babel under a Python 3.7 environment.

Given a simple example to demonstrate how the algorithm works, we assume there are only two reference compounds, *i* and *j*, whose docking scores are − 8.0 and − 10.0 kcal/mol and their experimental values are − 7.0 and − 8.0 kcal/mol, respectively. The docking score of the query compound is − 9.0 and the similarity between the query compound and reference compound *i* and *j* are 0.9 and 0.5, respectively. Assuming p is 4, then after the calibration, the new docking score for the query compound becomes:

$${DS}_{cali}=-9.0\frac{1}{{0.9}^{4}+{0.5}^{4}}\left[{0.9}^{4}\frac{-7.0}{-8.0}+{0.5}^{4}\frac{-8.0}{-10.0}\right]=-7.82$$

Apparently, reference compound *i* has more impact than *j* in the docking score calibration for this query compound. It is noted that our algorithm may not always improve the performance of docking score. There is a possibility that the docking score becomes worse after the calibration. However, we expect the similarity-based calibration can improve the binding affinity prediction in most scenarios with a certain size of the reference set.

### Performance evaluation

To reduce the systematic error during the calculation, the random separation of compounds into training and testing sets before the calibration was repeated for ten times for each target. The mean value and 95 % CI for all performance metrics were then calculated. To evaluate the scoring and ranking performance of our algorithm, for each receptor, the docking score of compounds in the test set was compared to their experimental energies individually utilizing four different measurements, RMSE, MAE, R^2^ and PI. By comparing the mean calibrated docking score with the original docking score, the scoring function was considered to be improved if RMSE and MAE reduced, while R^2^ and PI increased. We also calculated the difference between the calibrated docking score and the original one. The difference is respectively represented by dRMSE, dMAE, dR^2^ and dPI. For the evaluation of screening power, the area under the curve (AUC) of receiver operating characteristic (ROC) curve and enrichment factor (EF) at 10 % (EF_10 %_) and 40 % (EF_40 %_) levels were adopted as the performance metrics. In comparison to the simple docking scoring function, our new calibration algorithm on docking score was considered to have better screening power if AUC and EF increased. We applied the same protocols to evaluate the performance of the calibration algorithm on the datasets of A2AR and CFX targets from the DUD-E database, except that the enrichment factors were calculated with different hit rates. Considering the sample sizes of datasets for these two targets are relatively large in the DUE-E database, we utilized EF_1 %_ and EF_10 %_ to evaluate the screening power. The equations for the calculation of metrics PI and EF were described in detail in Supplementary Information.

We defined two scenarios, “focus library” and “diverse library”, which are respectively appliable to drug lead optimization and lead identification in drug discovery, to evaluate the algorithm by limiting the range of Tc. In other words, the training compounds which did not meet the criterion of Tc range were excluded from calculations for the calibration. In the “focus library” scenario, for each system, we set up a lower bound Tc value. Below this threshold, Tc will be too low to improve the performance of the scoring function. In the “diverse library” scenario, besides the lower bound Tc value, we also set up an upper bound for Tc. We randomly collected 10,000 screening compounds from ZINC database [27] (https://zinc.docking.org/) and calculated the structural similarity between testing compounds and screening compounds individually. The Tc value at which more than a half of screening compounds are lower than is selected as the upper bound Tc value. It is noted that this is a very stringent method to determine the Tc upper bound value.

## Results and discussion

### The impact of fingerprint and CSE function

**Fig. 2:**
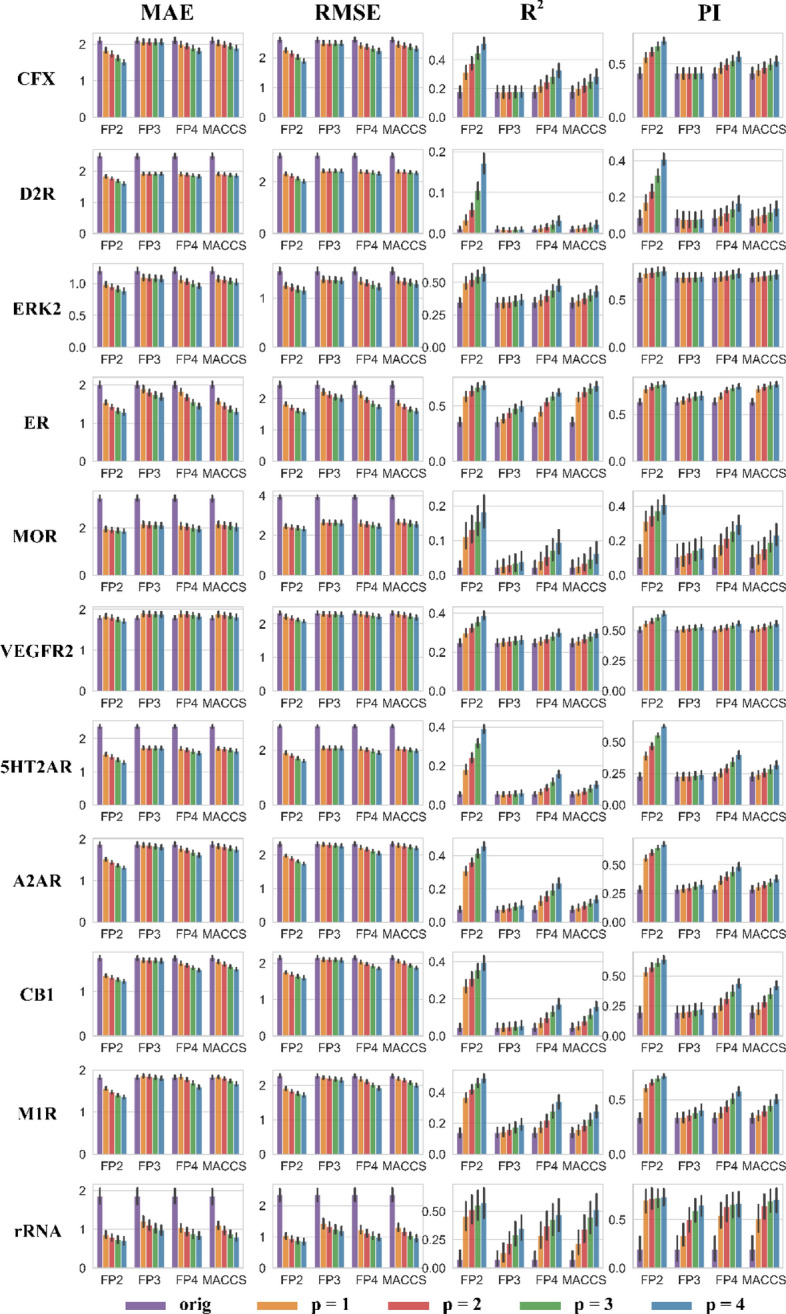
The comparison of RMSE, MAE, R^2^ and PI values before and after the calibration for 11 drug receptors under the conditions of different fingerprint type and chemical similarity effect function. “*orig*” refers to original docking scores before the calibration

We first studied the calibration performance using the Glide scoring function. For all 11 systems, the scoring power measured by RMSE and MAE and ranking power measured by R^2^ and PI of the original docking scoring function and the hybrid scoring functions applying different fingerprints and CSE function are shown in Additional file 1: Tables S3, S4 and Fig. 2. According to Additional file 1: Tables S3, S4, the developed algorithm can improve the accuracy of original docking score for most of systems, no matter what fingerprint was used or what CSE function was adopted. Specifically, when FP2 fingerprint was used for the similarity calculation between compounds in our algorithm, the docking scores can improve, regardless of types of systems or CSE function. Similarly, when ${CSE=S}_{ij}^{4}$, the performance of the scoring function enhanced for all 4 fingerprints. The comparison among the performance of the algorithms when employing different fingerprints and CSE functions is clearly illustrated in Fig. 2. For most systems, the enhanced effect of the algorithms with different calibrating functions can be ranked from the largest to the smallest when the p value varied from 4 to 1. As to fingerprint type, Fig. 2 also demonstrated that FP2 stands out as it mostly has lower RMSE and MAE, higher R^2^ and PI than other fingerprints.

To test the generalizability of our calibration algorithm, we also studied the calibration performance using docking scores generated by another commonly-used docking program, AutoDock Vina. As shown in Additional file 1: Table S5 and Figure S1, the same conclusion was reached for this docking program, i.e., the S^4^ outperforms other CES functions and FP2 outperforms other fingerprint types.

It is easy to understand why the performance of the algorithm became better when p value increased. As p value rises, the impact of the reference compounds that are structurally similar to the query compounds increases, meanwhile the impact of the reference compounds with low similarity reduces. As such, the weight of the similarity contribution will boost if the power of the similarity increases in the formula. It can be expected if the p value is higher than 4, the algorithm might continue improving the scoring function even in a more positive way. However, to balance the contributions from both the original docking scores and compound similarity effect, we let the maximal p value stop at 4.

Another interesting factor that can affect the performance of the algorithm is the type of fingerprints. The different underlying mechanisms of those fingerprints are likely to explain their different effects. Unlike FP3, FP4 and MACCS that are substructure-based fingerprints based on sets of SMARTS patterns, FP2 is a path-based fingerprint that indexes small molecules fragments based on linear segments of up to 7 atoms, which might elucidate why FP2 performed better in our algorithm [28]. As FP2 is more specific and can be used in any initial chemical searches, we assume the similarity calculation based on FP2 is able to amplify the weights of those structurally similar references to a greater extent and better offset the shortage of traditional scoring function. For example, the traditional docking score is always averagely high even for those ligands with relatively low binding affinity, leading to insufficient differentiation of docking results. On the other hand, the different performances of those three substructure-based fingerprints (FP3, FP4 and MACCS), are likely caused by more complicated reasons. One probable reason is their different number of descriptors. For example, utilizing FP3 improved the scoring function very limitedly, which might be explained by its limited number of bits of 55 versus FP4 with 307 bits and MACCS with 166 bits stored in Open Babel [29].

The calibration performance using the best hybrid function is similar for the two docking scoring functions. As shown in Table 2, for the tested 11 receptors with related thousands of ligands, on average, MAE decreased from 2.05 (Glide) to 1.34, 1.74 (AutoDock Vina) to 1.15; RMSE decreased from 2.53 (Glide) to 1.69, 2.11 (AutoDock Vina) to 1.47; while R^2^ increased from 0.14 (Glide) to 0.44, and from 0.16 (AutoDock Vina) to 0.50; PI increased from 0.34 (Glide) to 0.66, and from 0.39 (AutoDock Vina) to 0.71. In other words, the improvement for average values of mean MAE, RMSE, R^2^ and PI over 11 receptors are 0.71 kcal/mol, 0.84 kcal/mol, 0.30, and 0.32 respectively for the Glide docking scoring function, and the corresponding values are 0.59 kcal/mol, 0.64 kcal/mol, 0.34 and 0.32 for the AutoDock Vina scoring function. Interestingly, the boost performance on scoring power (RMSE and MAE) is better for Glide docking scoring function, while the ranking power (R^2^ and PI) is better for AutoDock Vina.

**Table 2:** Mean RMSE (kcal/mol), MAE (kcal/mol), R^2^ and PI before and after calibration using the best hybrid scoring function (FP2 fingerprint with CSE = S^4^) for 11 receptors

Receptor	Glide								AutoDock Vina							
	MAE		RMSE		R^2^		PI		MAE		RMSE		R^2^		PI	
	*cali*	*orig*	*cali*	*orig*	*cali*	*orig*	*cali*	*orig*	*cali*	*orig*	*cali*	*orig*	*cali*	*orig*	*cali*	*orig*
CFX	1.50	2.10	1.89	2.60	0.52	0.18	0.72	0.41	1.40	1.96	1.76	2.35	0.50	0.12	0.72	0.33
D2R	1.60	2.49	2.03	3.02	0.17	0.01	0.41	0.09	1.35	1.85	1.71	2.23	0.26	0.03	0.52	0.14
ERK2	0.88	1.20	1.16	1.55	0.57	0.35	0.81	0.74	0.67	1.48	0.89	1.78	0.70	0.11	0.87	0.54
ER	1.28	2.01	1.59	2.45	0.69	0.36	0.82	0.63	1.30	1.76	1.70	2.17	0.51	0.14	0.71	0.36
MOR	1.87	3.28	2.33	3.94	0.18	0.02	0.41	0.11	1.42	1.73	1.78	2.10	0.38	0.10	0.64	0.31
VEGFR2	1.71	1.80	2.08	2.33	0.39	0.25	0.64	0.51	1.02	1.43	1.32	1.80	0.67	0.42	0.83	0.66
5HT2AR	1.28	2.38	1.61	2.88	0.39	0.05	0.63	0.23	1.27	1.59	1.62	1.98	0.32	0.11	0.58	0.36
A2AR	1.32	1.88	1.74	2.32	0.46	0.08	0.68	0.29	1.30	1.89	1.70	2.33	0.44	0.03	0.67	0.14
CB1	1.23	1.76	1.59	2.15	0.39	0.04	0.64	0.20	1.12	1.99	1.41	2.38	0.49	0.15	0.71	0.39
M1R	1.36	1.83	1.72	2.27	0.49	0.14	0.72	0.33	1.18	1.62	1.48	1.99	0.58	0.31	0.77	0.54
rRNA	0.70	1.86	0.85	2.35	0.58	0.08	0.74	0.19	0.63	1.88	0.80	2.10	0.70	0.26	0.78	0.48
Average	1.34	2.05	1.69	2.53	0.44	0.14	0.66	0.34	1.15	1.74	1.47	2.11	0.50	0.16	0.71	0.39

“*cali*” and “*orig*” represent the calibrated and original docking scores, respectively

**Fig. 3:**
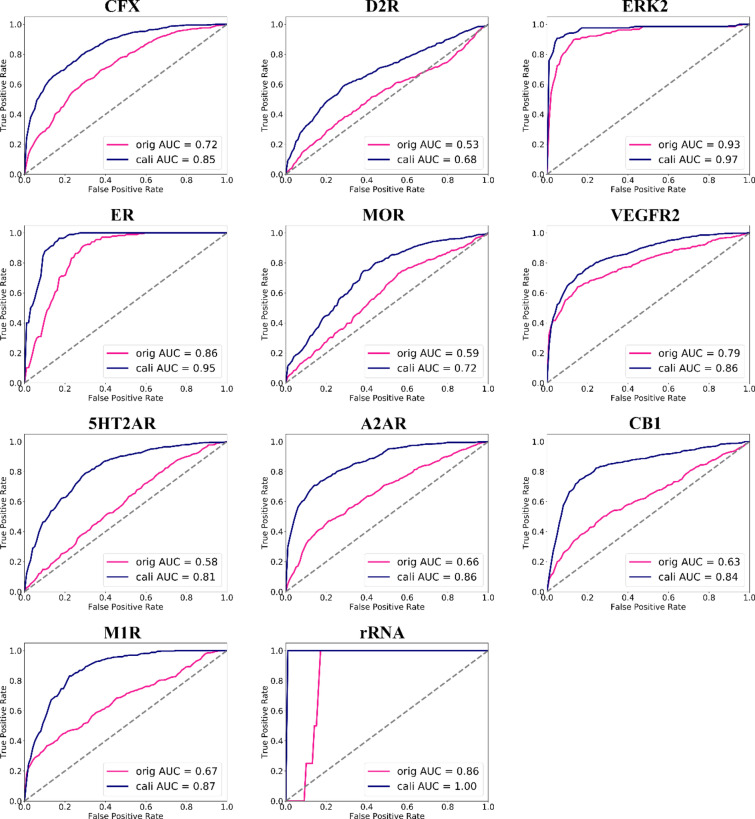
The mean ROC curves of screening results before and after calibration of Glide docking scores using the best hybrid scoring function (FP2 fingerprint with CSE = S^4^) for 11 receptors. “*cali*” and “*orig*” represent the calibrated and original docking scores, respectively

On the other hand, the improvement of performance on screening power by using our calibration algorithm further validated our approach. As shown in Table 3, EF_10 %_ and EF_40 %_ of the docking results after the calibration are better than the results before the calibration for all the scenarios except for EF_10 %_ of VEGFR2, for which the value before the calibration is slightly better (2.18 vs. 2.15). Considering the small sample size for rRNA receptor, it is reasonable to find significant larger enrichment factor values in the calculated metrics. After excluding the rRNA target, on average the mean EF_10 %_ and EF_40 %_ increased about 25 % and 22 % after the calibration of docking scores. Similarly, mean AUC of the docking results after the calibration is significantly improved for all the targets, as shown in Table 3 and illustrated in Fig. 3. Without considering the performance on rRNA target, on average the AUC improved approximately 20 % for the docking results after the calibration. Of note that the measurement of screening power may be biased for some drug target due to the imbalance between the number of actives and inactives, such as the actives only account for about 12 % of the total compounds in the test sets.

**Table 3:** Mean AUC, EF_10 %_ and EF_40 %_ of screening results before and after calibration of Glide docking scores using the best hybrid scoring function (FP2 fingerprint with CSE = S^4^) for 11 receptors

Receptor	EF_10 %_		EF_40 %_		AUC		Actives	Inactives
	*cali*	*orig*	*cali*	*orig*	*cali*	*orig*		
CFX	2.24 (0.13)	1.85 (0.15)	1.72 (0.08)	1.46 (0.08)	0.85 (0.02)	0.72 (0.03)	84 (6)	62 (3)
D2R	1.96 (0.31)	1.34 (0.15)	1.48 (0.14)	1.14 (0.12)	0.68 (0.05)	0.53 (0.05)	55 (4)	104 (6)
ERK2	7.73 (0.43)	6.26 (0.41)	2.44 (0.07)	2.40 (0.09)	0.97 (0.02)	0.93 (0.02)	20 (2)	151 (7)
ER	2.67 (0.25)	1.91 (0.42)	2.24 (0.09)	1.90 (0.16)	0.95 (0.01)	0.86 (0.03)	18 (3)	32 (4)
MOR	1.52 (0.33)	1.19 (0.31)	1.36 (0.13)	1.13 (0.10)	0.72 (0.05)	0.59 (0.04)	45 (4)	51 (8)
VEGFR2	2.15 (0.12)	2.18 (0.10)	1.75 (0.06)	1.64 (0.06)	0.86 (0.03)	0.79 (0.03)	59 (6)	70 (4)
5HT2AR	1.81 (0.15)	1.20 (0.18)	1.54 (0.08)	1.13 (0.04)	0.81 (0.02)	0.58 (0.02)	77 (5)	85 (5)
A2AR	2.45 (0.13)	1.66 (0.22)	1.87 (0.06)	1.37 (0.05)	0.86 (0.01)	0.66 (0.02)	67 (5)	109 (6)
CB1	2.09 (0.15)	1.67 (0.21)	1.83 (0.08)	1.32 (0.12)	0.84 (0.02)	0.63 (0.03)	54 (4)	76 (4)
M1R	2.39 (0.24)	2.33 (0.28)	1.90 (0.08)	1.33 (0.11)	0.87 (0.02)	0.67 (0.03)	42 (5)	73 (6)
rRNA	11.50 (2.99)	0.00	2.59 (0.20)	2.59 (0.20)	1.00	0.86 (0.03)	1	13 (2)

“*cali*” and “*orig*” represent the calibrated and original docking scores, respectively. The average numbers of compounds allocated in the active and inactive sets are shown in the table. 95 % CI for each metrics is displayed in the parenthesis

### The impact from receptor categories on the calibration performance

**Fig. 4:**
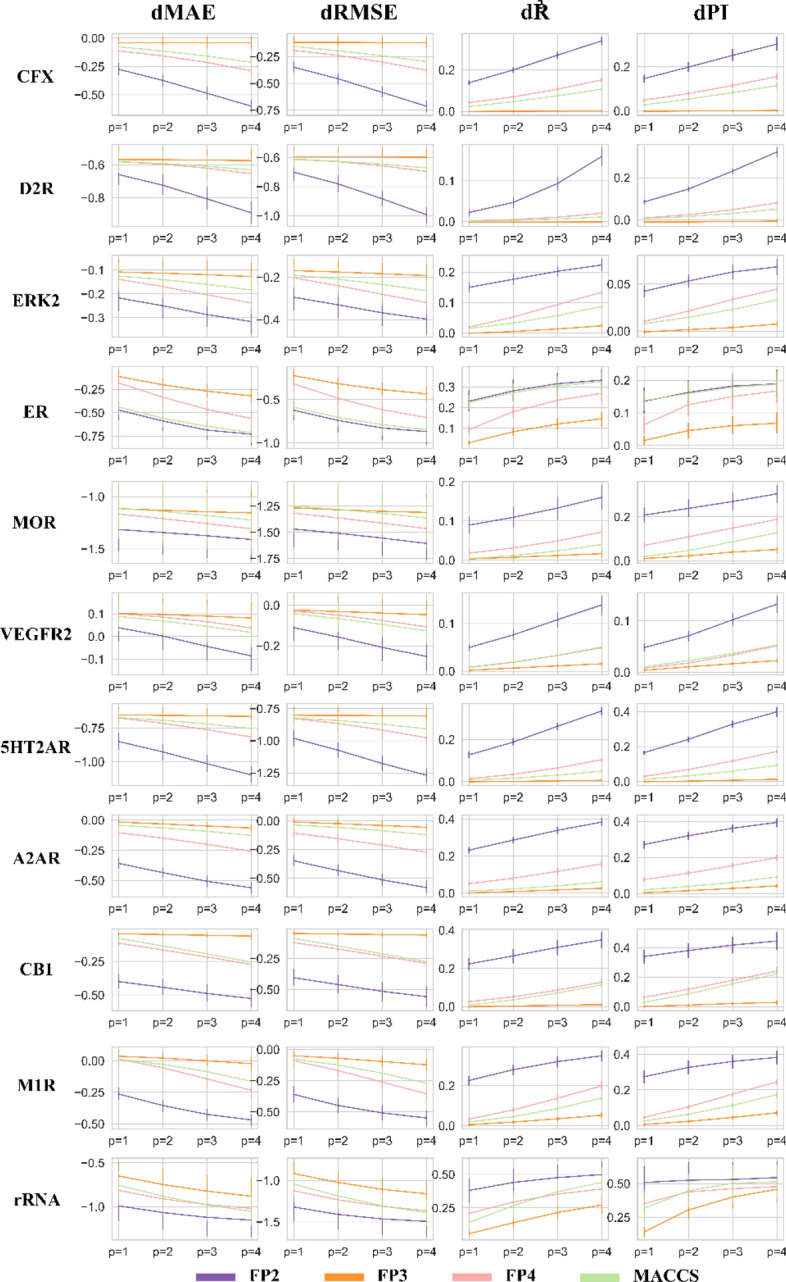
The changes of RMSE, MAE, R^2^ and PI values between the calibrated docking scores and the original docking scores using the Glide docking scoring function under the conditions of different fingerprint type and chemical similarity effect function, *S*^*p*^, where p takes a value of 1, 2, 3 or 4

As shown in Table 2; Fig. 2 and Additional file 1: Figure S1, the basic performance of docking score for all systems is different. Take the Glide docking scoring function as an example, for ERK2 drug target, the performance of original docking results is acceptable with a low mean MAE (1.20 kcal/mol) and RMSE (1.55 kcal/mol), and a relatively high mean R^2^ (0.35) and PI (0.74). On the other hand, for some receptors such as MOR, the performance of the original docking results is not satisfying with a high mean MAE (3.28 kcal/mol) and RMSE (3.94 kcal/mol), and a low mean R^2^ (0.02) and PI (0.11). Therefore, in order to further compare the improved effect of the algorithm between different systems by excluding the impact from initial baselines of various systems, we quantitatively estimated the difference between the calibrated docking score and original docking score using parameters dRMSE, dMAE, dR^2^ and dPI which quantitively measure the difference of those measurements before and after the calibration (Additional file 1: Table S4 and Fig. 4). It is observed that the extent of the improvement by using this algorithm varied from system to system, which indicates that although the developed algorithm can be adaptable for various receptors, its enhanced effect can still differ and depends on the receptor to some extent. The improvement on the scoring power and ranking power is more prominently for those systems with poor docking performance, such as D2R, MOR, 5HT2AR, and CB1. After the calibration using the best hybrid scoring function, the PI increases by 356 %, 273 %, 174 % and 220 % for the four systems correspondingly (Table 2). After the calibration, not only the docking performance is enhanced, but also the standard deviations and the CIs of the metrics measuring the docking performance are decreased among different drug receptors.

### Application of calibration in drug lead identification

In above, we discussed our hybrid scoring function can enhance screening performance for focused compound libraries in drug lead optimization. Next, we evaluated how well the best hybrid function (FP2 fingerprint and CSE = S^4^) performs for diverse compound libraries in drug lead identification using two studies. First, we created “diverse libraries” by imposing an upper limit of Tc as described in Methods section. When we calibrated the docking score of a query compound, we applied an upper limit of Tc value to exclude reference compounds which are structurally similar to the query compound to participate calibration. Encouragingly, even applying relatively low upper bound Tc values, our best hybrid scoring function can still enhance the docking performance for all receptors as shown in Table 4, even though the extend of the enhancement becomes much smaller as expected. The average values of mean MAE and RMSE decreased by 14.1 %, 14.7 %, respectively; and R^2^ and PI increased by 26.7 % and 17.1 %, respectively, after the calibration. In a real situation, the enhancement may be significantly larger as demonstrated in the second study.

**Table 4:** Mean RMSE (kcal/mol), MAE (kcal/mol), R^2^ and PI before and after calibration of Glide docking scores for 11 receptors by giving similarity a range with lower and upper bound in diverse library

Receptor	Tc range	MAE		RMSE		R^2^		PI	
		*Cali*	*Orig*	*Cali*	*Orig*	*Cali*	*Orig*	*Cali*	*Orig*
CFX	[0.30,0.45]	1.96 (0.06)	2.01 (0.07)	2.40 (0.07)	2.52 (0.07)	0.20 (0.04)	0.20 (0.04)	0.46 (0.04)	0.45 (0.04)
D2R	[0.30,0.40]	1.92 (0.05)	2.47 (0.05)	2.41 (0.07)	3.01 (0.06)	0.01 (0.01)	0.01 (0.01)	0.10 (0.04)	0.09 (0.04)
ERK2	[0.30,0.40]	1.09 (0.03)	1.19 (0.04)	1.38 (0.07)	1.54 (0.05)	0.43 (0.05)	0.36 (0.04)	0.77 (0.04)	0.76 (0.04)
ER	[0.20,0.35]	1.79 (0.09)	2.11 (0.08)	2.15 (0.07)	2.53 (0.06)	0.43 (0.06)	0.37 (0.05)	0.69 (0.05)	0.65 (0.04)
MOR	[0.35,0.40]	2.19 (0.10)	3.11 (0.15)	2.70 (0.10)	3.77 (0.17)	0.07 (0.02)	0.03 (0.02)	0.23 (0.06)	0.14 (0.07)
VEGFR2	[0.25,0.40]	1.89 (0.08)	1.78 (0.08)	2.34 (0.11)	2.33 (0.09)	0.25 (0.05)	0.24 (0.04)	0.51 (0.05)	0.49 (0.05)
5HT2AR	[0.30,0.45]	1.69 (0.07)	2.36 (0.09)	2.09 (0.08)	2.87 (0.09)	0.09 (0.04)	0.07 (0.03)	0.25 (0.06)	0.24 (0.08)
A2AR	[0.35,0.40]	1.84 (0.08)	1.84 (0.09)	2.29 (0.08)	2.27 (0.09)	0.13 (0.03)	0.09 (0.03)	0.36 (0.05)	0.29 (0.06)
CB1	[0.25,0.40]	1.65 (0.05)	1.78 (0.05)	2.08 (0.05)	2.19 (0.06)	0.09 (0.03)	0.04 (0.02)	0.30 (0.06)	0.21 (0.06)
M1R	[0.35,0.40]	1.80 (0.10)	1.83 (0.07)	2.20 (0.12)	2.25 (0.08)	0.24 (0.04)	0.13 (0.03)	0.47 (0.04)	0.32 (0.04)
rRNA	[0.30,0.35]	1.64 (0.11)	1.78 (0.23)	1.93 (0.11)	2.24 (0.25)	0.14 (0.06)	0.11 (0.06)	0.41 (0.10)	0.22 (0.18)
Average		1.77	2.02	2.18	2.50	0.19	0.15	0.41	0.35

“*Cali*” and “*Orig*” represent the calibrated and original docking scores, respectively. 95 % CI for each metrics is displayed in the parenthesis

In the second study, we recalculated docking scores of the Glide scoring function for a set of external test compound libraries collected by DUDE-E database for two drug targets, A2AR and CFX. Unlike the first study, we did not impose an upper bound of Tc in selecting reference compounds to mimic the real situation in virtual screening studies, however, for the test compound libraries, we eliminated all the entries which were duplicated with reference compounds. The performance of the best hybrid docking scoring function is summarized in Additional file 1: Tables S6, S7 and shown in Additional file 1: Figures S2, S3. The MAE and RMSE were respectively dropped from 1.44 to 1.05, and 1.78 to 1.37 kcal/mol for A2AR; and the two scoring power metrics were decreased from 2.71 to 1.66 and 3.25 to 2.13 kcal/mol for CFX. Similarly, the ranking power metrics R^2^ and PI were also significantly increased for both systems. For A2AR, R^2^ changed from 0.05 to 0.19 and PI changed from 0.24 to 0.46 (a 92 % increase); and for CFX, R^2^ changed from 0.02 to 0.16, and PI changed from 0.14 to 0.42 (a 200 % increase). As for the screening power, the EF_1 %_ and EF_10 %_ respectively enhanced from 1.18 to 1.35 and from 1.12 to 1.32 for CFX, while these two metrics correspondingly increased from 0.98 to 1.06 and 1.13 to 1.28 for A2AR. Last, the AUC values were increased from 0.58 to 0.71 for CFX and from 0.61 to 0.71 for A2AR.

Taken together, in the scenario of drug lead identification, our calibration algorithm can still significantly improve the docking performance measured by MAE and RMSE for scoring power, R^2^ and PI for the ranking power and EF and AUC for screening power. On the other hand, we pointed out that our method is based on docking results, hence the final performance on ranking compounds after our calibration algorithm may not meet the high standards of correctly ranking and prioritizing top compounds in the next stage of lead optimization, for which the more rigorous but much more expensive methods, such as alchemical free energy calculation using free energy perturbation [9] and thermodynamic integration [30, 31], are usually adopted.

## Conclusions

In summary, we developed a novel algorithm for quickly improving the scoring power and ranking power of a general scoring function used in a docking program by calibrating the docking score according to the structural similarities between the query compound and a set of reference compounds, whose experimental binding affinities have been measured. To evaluate the performance of the algorithm, we collected 11 receptors from different categories and estimated the enhanced effect of our algorithm on the Glide docking score by utilizing merit metrics of RMSE, MAE, R^2^, PI, EF and AUC. Fingerprint type and structural similarity effect function, the two factors which can significantly impact the performance of the calibration algorithm were systematically explored. The results showed that our algorithm could enhance the performance of the original docking scoring function in both the focused-library and diverse-library scenarios. We found that a combination of using FP2 fingerprint and a S^4^ CSE function can maximize the calibration performance for most systems. For the scenario of using the hybrid scoring function in drug lead optimization, the PI increased by 0.32 for both the Glide and AutoDock Vina scoring functions; for the scenario of using the hybrid scoring function in drug lead identification, the PI values of docking screenings using external test sets increased by 0.22 and 0.28 for A2AR and CFX systems, respectively. Thus, we successfully developed an algorithm which integrates structure-based docking scores and ligand-based structural similarity scores into a hybrid scoring function and make a good use of known experimental values. With more and more measured binding affinity data collected by public databases like ChEMBL, our calibration algorithm could have more and more broad applications in structure-based drug design. Afterall, the significantly enhanced performance is achieved by a simple calibration algorithm whose computational cost is neglectable.

## Supplementary Information

**Additional file 1: Table S1.** Lists the name, entry code, resolution, released date and deposition author for each receptor studied in this paper. **Table S3.** Lists the RMSE, MAE, R^2^ and PI values before and after calibration of the Glide docking scores under the conditions of different CSE function and fingerprint. **Table S4.** Lists the difference of metrics for the measurement of docking performance before and after the calibration, i.e., dRMSE, dMAE, dR^2^ and dPI for the Glide scoring function. **Table S5.** Lists and **Figure S1.** Shows the RMSE, MAE, R^2^ and PI values before and after calibration of the AutoDock Vina docking scores under the conditions of different CSE functions and fingerprints. **Table S6.** Shows RMSE, MAE, R^2^ and PI values before and after calibration of Glide docking scores for compounds in the external test sets from DUD-E database. **Figure S2.** Shows the comparison of RMSE, MAE, R2 and PI values before and after the calibration of the Glide docking scores for A2AR and CFX external test sets using the best hybrid scoring function (FP2 fingerprint with CSE = S4). **Table S7.** Displays AUC, EF_1 %_ and EF_10 %_ values before and after calibration of Glide docking scores for compounds in the external test sets from DUD-E database. **Figure S3.** Shows ROC curves before and after calibration of the Glide docking scores for A2AR and CFX external test sets.

**Additional file 2: Table S2A.** Glide docking scores (kcal/mol) and experimental energies (kcal/mol) of selected compounds for all 11 targets in reference set and validation set.

**Additional file 3: Table S2B.** AutoDock Vina docking scores (kcal/mol) and experimental energies (kcal/mol) of selected compounds for all 11 targets in reference set and validation set.

## Data Availability

All data come from publication domain. The associated parameters of the hybrid scoring functions for each drug target were presented in tables.
