# Supplementary material for: Incorporating structural similarity into a scoring function to enhance the prediction of binding affinities
Source: J Cheminform. 2021 Feb 15;13:11. doi: 10.1186/s13321-021-00493-4 (PMC7884591; doi:10.1186/s13321-021-00493-4)
Supplement: Supplementary file 1 — Additional file 1: Table S1. Lists the name, entry code, resolution, released date and deposition author for each receptor studied in this paper. Table S3. Lists the RMSE, MAE, R2 and PI values before and after calibration of the Glide docking scores under the conditions of different CSE function and fingerprint. Table S4. Lists the difference of metrics for the measurement of docking performance before and after the calibration, i.e., dRMSE, dMAE, dR2 and dPI for the Glide scoring function. Table S5. Lists and Figure S1. Shows the RMSE, MAE, R2 and PI values before and after calibration of the AutoDock Vina docking scores under the conditions of different CSE functions and fingerprints. Table S6. Shows RMSE, MAE, R2 and PI values before and after calibration of Glide docking scores for compounds in the external test sets from DUD-E database. Figure S2. Shows the comparison of RMSE, MAE, R2 and PI values before and after the calibration of the Glide docking scores for A2AR and CFX external test sets using the best hybrid scoring function (FP2 fingerprint with CSE = S4). Table S7. Displays AUC, EF1 % and EF10 % values before and after calibration of Glide docking scores for compounds in the external test sets from DUD-E database. Figure S3. Shows ROC curves before and after calibration of the Glide docking scores for A2AR and CFX external test sets. [file 13321_2021_493_MOESM1_ESM.docx]

**Additional Information for:**

**Incorporating structural similarity into a scoring function to enhance the prediction of binding affinities**

*Beihong Ji, Xibing He, Yuzhao Zhang, Jingchen Zhai, Viet Hoang Man, Shuhan Liu, Junmei Wang**

* To whom correspondence should be addressed: junmeiwang@pitt.edu

**Matrices for Evaluation of Screening Performance**

- **Enrichment Factor (EF)**: The proportion of the true active binders in the sampled subset relative to the proportion of true active binders in the total dataset.

$${EF}_{x\%}=\frac{{NTB}_{s}}{N_{s}}/\frac{{NTB}_{t}}{N_{t}}$$

Where ${NTB}_{s}$ is the number of true active binders in the sampled subset, ${NTB}_{t}$ is the total number of true active binders in the total dataset, $N_{s}$ is the number of compounds in the subset and $N_{t}$ is the number of compounds in the total dataset. x% is the proportion of top candidate selected from the ranked database.

- **Predictive Index (PI)**: A metric proposed by Pearlman and Charifson to measure the quality of a rank-ordering by the potency of a series of ligands.

$$PI= \frac{\sum_{j>i} \sum_{i} w_{ij}C_{ij}}{\sum_{j>i} \sum_{i} w_{ij}}$$

$$w_{ij}=\left| E\left( j \right)-E(i) \right|$$

$$C_{ij}=\left\{ \begin{aligned} +1 if \frac{E\left( j \right)-E(i)}{P\left( j \right)-P(i)}<0 \\ +1 if \frac{E\left( j \right)-E(i)}{P\left( j \right)-P(i)}>0 \\ 0 if P\left( j \right)-P\left( i \right)=0 \end{aligned} \right.$$

Where $E(i)$ and $P(i)$ are the experimental and predicted binding free energies of compound *i*. The *PI* ranges from -1 to +1, depending on how well the predicted ranking matches the experimental ordering. A value of +1 indicates perfect predictions, a value of -1 indicates predictions that are perfectly anticorrelated, and a value of 0 arises from random results. In essence, the method considers each pair of compounds *i* and *j* in turn. Large differences in binding free energies have a large weight, $w_{ij}$, which provides a large positive contribution to the final *PI*, if the rank-ordering of the pair is correct. Conversely, if *i* and *j* have a small difference in measured binding affinity, an incorrect prediction of the most potent binder has a minor impact on the final *PI*.

**Table S1.** Entry codes, resolutions, released dates and deposition authors for 11 receptors in Protein Data Bank (<https://www.rcsb.org/>).

| Receptor | Entry code | Resolution | Released date | Deposition Authors |
| --- | --- | --- | --- | --- |
| CFX | 1NFU | 2.05 Å | 2003-02-25 | Maignan, S., Guilloteau, J.P. |
| D2R | 6CM4 | 2.87 Å | 2018-03-14 | Wang, S., Che, T., etc |
| ERK2 | 6SLG | 1.33 Å | 2019-11-20 | Breed, J., Phillips, C. |
| ER | 3OS8 | 2.03 Å | 2010-11-10 | Bruning, J., Parent, A.A., etc |
| MOR | 5C1M | 2.10 Å | 2015-08-05 | Huang, W.J., Manglik, A., etc |
| VEGFR2 | 2OH4 | 2.05 Å | 2007-09-18 | Nolte, R.T. Wang, L. |
| 5HT2AR | 6A93 | 3.00 Å | 2019-02-13 | Kimura, T.K., Asada, H., etc |
| A2AR | 3EML | 2.60 Å | 2008-10-14 | Jaakola, V.-P., Griffith, M.T., etc |
| CB1 | 5XRA | 2.80 Å | 2017-07-12 | Hua, T., Vemuri, K., etc |
| M1R | 5CXV | 2.70 Å | 2016-03-09 | Sun, B., Feng, D., etc |
| rRNA | 2F4S | 2.80 Å | 2006-05-02 | Murray, J.B. Meroueh, S.O., etc |

**Table S3**. Mean RMSE (kcal/mol), MAE (kcal/mol), R^2^ and PI values before and after calibration of Glide docking scores for all receptors under the conditions of different fingerprint type and *p* value in CSE function (*S^p^*). “orig” refers to original, representing the initial situation before the calibration. 95% CI for each metrics is displayed in the parenthesis.

| **Function** | **MAE** | | | | **RMSE** | | | | **R^2^** | | | | **PI** | | | |
| --- | --- | --- | --- | --- | --- | --- | --- | --- | --- | --- | --- | --- | --- | --- | --- | --- |
|  | **fp2** | **fp3** | **fp4** | **maccs** | **fp2** | **fp3** | **fp4** | **maccs** | **fp2** | **fp3** | **fp4** | **maccs** | **fp2** | **fp3** | **fp4** | **maccs** |
| **CFX (Tc > 0.30)** | | | | | | | | | | | | | | | | |
| **orig** | 2.10 (0.09) | | | | 2.60 (0.09) | | | | 0.18 (0.05) | | | | 0.41 (0.06) | | | |
| **p=1** | 1.83 (0.09) | 2.06 (0.09) | 1.99 (0.08) | 2.03 (0.09) | 2.25 (0.10) | 2.49 (0.10) | 2.41 (0.10) | 2.45 (0.10) | 0.31 (0.06) | 0.18 (0.05) | 0.22 (0.05) | 0.20 (0.05) | 0.56 (0.05) | 0.41 (0.06) | 0.46 (0.06) | 0.44 (0.06) |
| **p=2** | 1.73 (0.09) | 2.06 (0.09) | 1.95 (0.08) | 1.99 (0.09) | 2.15 (0.09) | 2.48 (0.10) | 2.36 (0.10) | 2.41 (0.10) | 0.38 (0.06) | 0.18 (0.05) | 0.25 (0.06) | 0.22 (0.05) | 0.61 (0.05) | 0.41 (0.06) | 0.49 (0.06) | 0.47 (0.06) |
| **p=3** | 1.62 (0.08) | 2.06 (0.09) | 1.89 (0.08) | 1.94 (0.09) | 2.02 (0.09) | 2.48 (0.10) | 2.30 (0.10) | 2.36 (0.10) | 0.45 (0.05) | 0.18 (0.05) | 0.28 (0.06) | 0.25 (0.06) | 0.67 (0.04) | 0.42 (0.06) | 0.53 (0.06) | 0.50 (0.06) |
| **p=4** | 1.50 (0.08) | 2.06 (0.09) | 1.82 (0.08) | 1.89 (0.09) | 1.89 (0.08) | 2.48 (0.10) | 2.23 (0.10) | 2.31 (0.10) | 0.52 (0.05) | 0.18 (0.05) | 0.33 (0.06) | 0.28 (0.06) | 0.72 (0.04) | 0.42 (0.06) | 0.57 (0.05) | 0.53 (0.06) |
| **D2R (Tc > 0.30)** | | | | | | | | | | | | | | | | |
| **orig** | 2.49 (0.09) | | | | 3.02 (0.09) | | | | 0.01 (0.01) | | | | 0.09 (0.09) | | | |
| **p=1** | 1.84 (0.05) | 1.93 (0.05) | 1.91 (0.05) | 1.91 (0.05) | 2.32 (0.07) | 2.43 (0.07) | 2.41 (0.07) | 2.41 (0.07) | 0.03 (0.01) | 0.01 (0.01) | 0.01 (0.01) | 0.01 (0.01) | 0.17 (0.05) | 0.08 (0.05) | 0.10 (0.05) | 0.09 (0.05) |
| **p=2** | 1.77 (0.05) | 1.92 (0.05) | 1.90 (0.05) | 1.90 (0.05) | 2.24 (0.07) | 2.43 (0.07) | 2.39 (0.07) | 2.39 (0.07) | 0.06 (0.02) | 0.01 (0.01) | 0.02 (0.01) | 0.01 (0.01) | 0.23 (0.05) | 0.08 (0.05) | 0.11 (0.05) | 0.10 (0.05) |
| **p=3** | 1.69 (0.05) | 1.92 (0.05) | 1.87 (0.05) | 1.88 (0.05) | 2.14 (0.06) | 2.42 (0.07) | 2.36 (0.07) | 2.37 (0.07) | 0.10 (0.03) | 0.01 (0.01) | 0.02 (0.01) | 0.02 (0.01) | 0.32 (0.04) | 0.08 (0.05) | 0.13 (0.05) | 0.12 (0.05) |
| **p=4** | 1.60 (0.05) | 1.92 (0.05) | 1.84 (0.06) | 1.86 (0.05) | 2.03 (0.06) | 2.42 (0.07) | 2.32 (0.07) | 2.35 (0.07) | 0.17 (0.03) | 0.01 (0.01) | 0.03 (0.01) | 0.02 (0.01) | 0.41 (0.04) | 0.08 (0.05) | 0.17 (0.05) | 0.14 (0.05) |
| **ERK2 (Tc > 0.30)** | | | | | | | | | | | | | | | | |
| **orig** | 1.20 (0.06) | | | | 1.55 (0.08) | | | | 0.35 (0.05) | | | | 0.74 (0.05) | | | |
| **p=1** | 0.98 (0.05) | 1.09 (0.06) | 1.06 (0.05) | 1.08 (0.05) | 1.26 (0.07) | 1.39 (0.07) | 1.35 (0.07) | 1.36 (0.07) | 0.50 (0.06) | 0.34 (0.05) | 0.36 (0.05) | 0.36 (0.05) | 0.78 (0.06) | 0.74 (0.05) | 0.75 (0.05) | 0.75 (0.05) |
| **p=2** | 0.95 (0.05) | 1.09 (0.06) | 1.03 (0.05) | 1.06 (0.05) | 1.22 (0.07) | 1.38 (0.07) | 1.31 (0.07) | 1.34 (0.07) | 0.52 (0.06) | 0.35 (0.05) | 0.40 (0.05) | 0.38 (0.05) | 0.79 (0.05) | 0.74 (0.05) | 0.76 (0.05) | 0.75 (0.05) |
| **p=3** | 0.91 (0.05) | 1.08 (0.06) | 1.00 (0.05) | 1.04 (0.05) | 1.19 (0.07) | 1.37 (0.07) | 1.27 (0.07) | 1.32 (0.07) | 0.55 (0.06) | 0.36 (0.05) | 0.44 (0.05) | 0.40 (0.05) | 0.80 (0.05) | 0.74 (0.06) | 0.77 (0.05) | 0.76 (0.05) |
| **p=4** | 0.88 (0.05) | 1.07 (0.06) | 0.96 (0.05) | 1.02 (0.05) | 1.16 (0.07) | 1.36 (0.07) | 1.23 (0.07) | 1.29 (0.07) | 0.57 (0.06) | 0.37 (0.05) | 0.48 (0.05) | 0.43 (0.05) | 0.81 (0.05) | 0.74 (0.05) | 0.78 (0.05) | 0.77 (0.05) |
| **ER (Tc > 0.20)** | | | | | | | | | | | | | | | | |
| **orig** | 2.01 (0.11) | | | | 2.45 (0.13) | | | | 0.36 (0.05) | | | | 0.63 (0.04) | | | |
| **p=1** | 1.54 (0.08) | 1.90 (0.11) | 1.83 (0.10) | 1.58 (0.09) | 1.83 (0.07) | 2.23 (0.11) | 2.14 (0.10) | 1.87 (0.09) | 0.59 (0.05) | 0.39 (0.05) | 0.45 (0.04) | 0.58 (0.05) | 0.77 (0.04) | 0.65 (0.04) | 0.70 (0.03) | 0.77 (0.04) |
| **p=2** | 1.43 (0.08) | 1.81 (0.10) | 1.68 (0.10) | 1.46 (0.09) | 1.71 (0.08) | 2.14 (0.11) | 1.97 (0.09) | 1.75 (0.09) | 0.64 (0.05) | 0.44 (0.05) | 0.54 (0.04) | 0.63 (0.05) | 0.80 (0.04) | 0.68 (0.04) | 0.76 (0.03) | 0.79 (0.04) |
| **p=3** | 1.33 (0.08) | 1.75 (0.10) | 1.55 (0.08) | 1.37 (0.09) | 1.63 (0.08) | 2.07 (0.11) | 1.84 (0.08) | 1.67 (0.08) | 0.67 (0.05) | 0.48 (0.05) | 0.59 (0.04) | 0.66 (0.05) | 0.82 (0.04) | 0.70 (0.05) | 0.79 (0.03) | 0.81 (0.04) |
| **p=4** | 1.28 (0.09) | 1.69 (0.10) | 1.45 (0.08) | 1.30 (0.08) | 1.59 (0.09) | 2.02 (0.11) | 1.75 (0.07) | 1.61 (0.09) | 0.69 (0.04) | 0.50 (0.05) | 0.63 (0.04) | 0.68 (0.05) | 0.82 (0.04) | 0.70 (0.05) | 0.80 (0.03) | 0.82 (0.03) |
| **MOR (Tc > 0.35)** | | | | | | | | | | | | | | | | |
| **orig** | 3.28 (0.15) | | | | 3.94 (0.12) | | | | 0.02 (0.02) | | | | 0.11 (0.08) | | | |
| **p=1** | 1.97 (0.13) | 2.17 (0.15) | 2.12 (0.16) | 2.17 (0.16) | 2.47 (0.13) | 2.67 (0.14) | 2.62 (0.15) | 2.68 (0.16) | 0.11 (0.05) | 0.03 (0.02) | 0.04 (0.03) | 0.03 (0.03) | 0.31 (0.07) | 0.12 (0.08) | 0.17 (0.07) | 0.12 (0.08) |
| **p=2** | 1.94 (0.13) | 2.15 (0.15) | 2.07 (0.15) | 2.14 (0.17) | 2.43 (0.13) | 2.65 (0.14) | 2.57 (0.14) | 2.66 (0.16) | 0.13 (0.05) | 0.03 (0.03) | 0.05 (0.03) | 0.03 (0.03) | 0.34 (0.07) | 0.13 (0.08) | 0.21 (0.07) | 0.15 (0.08) |
| **p=3** | 1.91 (0.12) | 2.14 (0.15) | 2.02 (0.15) | 2.10 (0.17) | 2.38 (0.12) | 2.64 (0.14) | 2.52 (0.14) | 2.62 (0.16) | 0.16 (0.05) | 0.03 (0.03) | 0.07 (0.04) | 0.05 (0.03) | 0.38 (0.07) | 0.14 (0.08) | 0.25 (0.07) | 0.19 (0.08) |
| **p=4** | 1.87 (0.11) | 2.13 (0.15) | 1.97 (0.14) | 2.06 (0.17) | 2.33 (0.11) | 2.63 (0.15) | 2.47 (0.13) | 2.57 (0.16) | 0.18 (0.06) | 0.04 (0.03) | 0.09 (0.04) | 0.06 (0.04) | 0.41 (0.07) | 0.16 (0.08) | 0.29 (0.07) | 0.23 (0.08) |
| **VEGFR2 (Tc > 0.25)** | | | | | | | | | | | | | | | | |
| **orig** | 1.80 (0.05) | | | | 2.33 (0.07) | | | | 0.25 (0.02) | | | | 0.51 (0.03) | | | |
| **p=1** | 1.84 (0.07) | 1.90 (0.08) | 1.90 (0.08) | 1.89 (0.08) | 2.22 (0.08) | 2.31 (0.08) | 2.30 (0.08) | 2.29 (0.08) | 0.30 (0.02) | 0.25 (0.02) | 0.26 (0.02) | 0.26 (0.02) | 0.55 (0.03) | 0.51 (0.03) | 0.51 (0.03) | 0.52 (0.03) |
| **p=2** | 1.80 (0.07) | 1.90 (0.08) | 1.88 (0.08) | 1.87 (0.08) | 2.17 (0.07) | 2.30 (0.08) | 2.28 (0.08) | 2.26 (0.08) | 0.32 (0.02) | 0.26 (0.02) | 0.27 (0.02) | 0.27 (0.02) | 0.58 (0.02) | 0.52 (0.03) | 0.52 (0.02) | 0.53 (0.03) |
| **p=3** | 1.75 (0.06) | 1.89 (0.08) | 1.86 (0.08) | 1.84 (0.08) | 2.12 (0.05) | 2.29 (0.08) | 2.25 (0.08) | 2.24 (0.08) | 0.36 (0.03) | 0.26 (0.02) | 0.28 (0.02) | 0.28 (0.02) | 0.61 (0.02) | 0.52 (0.03) | 0.54 (0.02) | 0.54 (0.02) |
| **p=4** | 1.71 (0.05) | 1.88 (0.08) | 1.84 (0.07) | 1.82 (0.08) | 2.08 (0.05) | 2.28 (0.08) | 2.22 (0.08) | 2.20 (0.08) | 0.39 (0.03) | 0.26 (0.02) | 0.30 (0.02) | 0.30 (0.03) | 0.64 (0.02) | 0.53 (0.03) | 0.56 (0.02) | 0.56 (0.03) |
| **5HT2AR (Tc > 0.30)** | | | | | | | | | | | | | | | | |
| **orig** | 2.38 (0.06) | | | | 2.88 (0.06) | | | | 0.05 (0.01) | | | | 0.23 (0.04) | | | |
| **p=1** | 1.53 (0.06) | 1.73 (0.06) | 1.7 (0.06) | 1.71 (0.06) | 1.90 (0.07) | 2.08 (0.07) | 2.06 (0.07) | 2.06 (0.07) | 0.18 (0.03) | 0.05 (0.01) | 0.07 (0.02) | 0.06 (0.02) | 0.39 (0.04) | 0.23 (0.04) | 0.26 (0.04) | 0.24 (0.04) |
| **p=2** | 1.45 (0.06) | 1.72 (0.06) | 1.66 (0.06) | 1.69 (0.06) | 1.81 (0.07) | 2.08 (0.07) | 2.02 (0.07) | 2.04 (0.07) | 0.24 (0.03) | 0.06 (0.01) | 0.09 (0.02) | 0.07 (0.02) | 0.47 (0.03) | 0.23 (0.04) | 0.30 (0.04) | 0.26 (0.04) |
| **p=3** | 1.36 (0.06) | 1.72 (0.06) | 1.62 (0.06) | 1.66 (0.06) | 1.71 (0.06) | 2.08 (0.07) | 1.97 (0.07) | 2.01 (0.07) | 0.32 (0.03) | 0.06 (0.02) | 0.12 (0.02) | 0.08 (0.02) | 0.56 (0.02) | 0.23 (0.04) | 0.34 (0.04) | 0.29 (0.04) |
| **p=4** | 1.28 (0.05) | 1.72 (0.06) | 1.56 (0.06) | 1.63 (0.06) | 1.61 (0.06) | 2.07 (0.07) | 1.90 (0.07) | 1.97 (0.07) | 0.39 (0.02) | 0.06 (0.02) | 0.16 (0.02) | 0.11 (0.02) | 0.63 (0.01) | 0.24 (0.04) | 0.40 (0.04) | 0.32 (0.04) |
| **A2AR (Tc > 0.35)** | | | | | | | | | | | | | | | | |
| **orig** | 1.88 (0.07) | | | | 2.32 (0.07) | | | | 0.08 (0.02) | | | | 0.29 (0.04) | | | |
| **p=1** | 1.52 (0.05) | 1.86 (0.07) | 1.77 (0.06) | 1.83 (0.07) | 1.98 (0.04) | 2.31 (0.06) | 2.22 (0.06) | 2.29 (0.06) | 0.31 (0.03) | 0.08 (0.02) | 0.13 (0.03) | 0.09 (0.02) | 0.56 (0.03) | 0.29 (0.04) | 0.36 (0.04) | 0.31 (0.03) |
| **p=2** | 1.44 (0.05) | 1.84 (0.07) | 1.73 (0.06) | 1.81 (0.07) | 1.89 (0.04) | 2.30 (0.06) | 2.17 (0.06) | 2.27 (0.06) | 0.36 (0.03) | 0.08 (0.02) | 0.16 (0.03) | 0.10 (0.02) | 0.61 (0.03) | 0.30 (0.03) | 0.40 (0.04) | 0.33 (0.03) |
| **p=3** | 1.37 (0.04) | 1.83 (0.07) | 1.68 (0.06) | 1.78 (0.07) | 1.81 (0.04) | 2.28 (0.06) | 2.11 (0.06) | 2.24 (0.06) | 0.41 (0.03) | 0.09 (0.02) | 0.19 (0.04) | 0.12 (0.02) | 0.65 (0.02) | 0.32 (0.03) | 0.44 (0.04) | 0.35 (0.03) |
| **p=4** | 1.32 (0.04) | 1.81 (0.07) | 1.62 (0.06) | 1.75 (0.07) | 1.74 (0.03) | 2.27 (0.06) | 2.05 (0.06) | 2.20 (0.06) | 0.46 (0.03) | 0.10 (0.03) | 0.23 (0.04) | 0.14 (0.03) | 0.68 (0.02) | 0.33 (0.04) | 0.49 (0.04) | 0.38 (0.03) |
| **CB1 (Tc > 0.30)** | | | | | | | | | | | | | | | | |
| **orig** | 1.76 (0.06) | | | | 2.15 (0.06) | | | | 0.04 (0.03) | | | | 0.20 (0.06) | | | |
| **p=1** | 1.36 (0.04) | 1.72 (0.06) | 1.65 (0.05) | 1.68 (0.05) | 1.75 (0.04) | 2.10 (0.06) | 2.03 (0.05) | 2.07 (0.05) | 0.27 (0.04) | 0.04 (0.03) | 0.07 (0.03) | 0.05 (0.03) | 0.54 (0.04) | 0.20 (0.06) | 0.26 (0.06) | 0.22 (0.05) |
| **p=2** | 1.32 (0.04) | 1.71 (0.06) | 1.60 (0.05) | 1.62 (0.05) | 1.69 (0.05) | 2.10 (0.06) | 1.98 (0.04) | 2.00 (0.05) | 0.31 (0.04) | 0.05 (0.03) | 0.10 (0.03) | 0.08 (0.03) | 0.58 (0.04) | 0.21 (0.06) | 0.31 (0.05) | 0.28 (0.05) |
| **p=3** | 1.27 (0.04) | 1.70 (0.06) | 1.54 (0.04) | 1.57 (0.04) | 1.64 (0.05) | 2.10 (0.06) | 1.92 (0.04) | 1.94 (0.04) | 0.35 (0.05) | 0.05 (0.03) | 0.13 (0.03) | 0.12 (0.03) | 0.61 (0.04) | 0.22 (0.06) | 0.37 (0.05) | 0.35 (0.05) |
| **p=4** | 1.23 (0.04) | 1.70 (0.06) | 1.49 (0.04) | 1.50 (0.04) | 1.59 (0.06) | 2.09 (0.06) | 1.86 (0.03) | 1.88 (0.04) | 0.39 (0.05) | 0.05 (0.03) | 0.17 (0.03) | 0.16 (0.03) | 0.64 (0.04) | 0.22 (0.06) | 0.44 (0.04) | 0.42 (0.04) |
| **M1R (Tc > 0.30)** | | | | | | | | | | | | | | | | |
| **orig** | 1.83 (0.06) | | | | 2.27 (0.06) | | | | 0.14 (0.04) | | | | 0.33 (0.05) | | | |
| **p=1** | 1.57 (0.04) | 1.87 (0.05) | 1.85 (0.05) | 1.68 (0.05) | 1.91 (0.06) | 2.22 (0.06) | 2.18 (0.06) | 2.07 (0.05) | 0.36 (0.04) | 0.15 (0.04) | 0.17 (0.04) | 0.05 (0.03) | 0.61 (0.03) | 0.34 (0.05) | 0.38 (0.06) | 0.22 (0.05) |
| **p=2** | 1.48 (0.04) | 1.85 (0.05) | 1.78 (0.05) | 1.62 (0.05) | 1.82 (0.06) | 2.20 (0.06) | 2.10 (0.06) | 2.00 (0.05) | 0.42 (0.03) | 0.16 (0.04) | 0.22 (0.05) | 0.08 (0.03) | 0.66 (0.03) | 0.36 (0.06) | 0.44 (0.06) | 0.28 (0.05) |
| **p=3** | 1.41 (0.04) | 1.83 (0.05) | 1.69 (0.05) | 1.57 (0.04) | 1.76 (0.06) | 2.17 (0.06) | 2.01 (0.06) | 1.94 (0.04) | 0.46 (0.03) | 0.17 (0.04) | 0.28 (0.05) | 0.12 (0.03) | 0.69 (0.02) | 0.38 (0.06) | 0.51 (0.05) | 0.35 (0.05) |
| **p=4** | 1.36 (0.04) | 1.81 (0.06) | 1.60 (0.06) | 1.50 (0.04) | 1.72 (0.06) | 2.15 (0.07) | 1.92 (0.07) | 1.88 (0.04) | 0.49 (0.03) | 0.19 (0.05) | 0.34 (0.05) | 0.16 (0.03) | 0.72 (0.02) | 0.40 (0.06) | 0.58 (0.05) | 0.42 (0.04) |
| **rRNA (Tc > 0.30)** | | | | | | | | | | | | | | | | |
| **orig** | 1.86 (0.24) | | | | 2.35 (0.25) | | | | 0.08 (0.08) | | | | 0.19 (0.15) | | | |
| **p=1** | 0.87 (0.12) | 1.21 (0.16) | 1.04 (0.13) | 1.10 (0.12) | 1.03 (0.12) | 1.43 (0.20) | 1.22 (0.15) | 1.30 (0.15) | 0.46 (0.15) | 0.13 (0.10) | 0.29 (0.14) | 0.22 (0.13) | 0.70 (0.15) | 0.33 (0.14) | 0.55 (0.16) | 0.51 (0.17) |
| **p=2** | 0.79 (0.11) | 1.11 (0.15) | 0.95 (0.13) | 0.98 (0.12) | 0.94 (0.12) | 1.32 (0.19) | 1.11 (0.14) | 1.16 (0.13) | 0.52 (0.16) | 0.22 (0.12) | 0.37 (0.16) | 0.34 (0.15) | 0.72 (0.14) | 0.50 (0.15) | 0.63 (0.16) | 0.64 (0.16) |
| **p=3** | 0.74 (0.11) | 1.03 (0.15) | 0.88 (0.13) | 0.88 (0.12) | 0.88 (0.12) | 1.24 (0.18) | 1.04 (0.13) | 1.04 (0.13) | 0.55 (0.16) | 0.29 (0.13) | 0.43 (0.17) | 0.45 (0.16) | 0.72 (0.12) | 0.59 (0.14) | 0.66 (0.16) | 0.70 (0.15) |
| **p=4** | 0.70 (0.11) | 0.98 (0.15) | 0.84 (0.12) | 0.80 (0.12) | 0.85 (0.12) | 1.19 (0.17) | 0.98 (0.12) | 0.96 (0.13) | 0.58 (0.16) | 0.35 (0.14) | 0.47 (0.17) | 0.52 (0.17) | 0.74 (0.11) | 0.65 (0.13) | 0.67 (0.16) | 0.71 (0.15) |

**Table S4.** The mean difference of RMSE (kcal/mol), MAE (kcal/mol), R^2^ and PI values between the original and calibrated Glide docking scores for all receptors under the conditions of different fingerprint type and *p* value in CSE function (*S^p^*). 95% CI for each metrics is displayed in the parenthesis.

| **Function** | **dMAE** | | | | **dRMSE** | | | | **dR^2^** | | | | **dPI** | | | |
| --- | --- | --- | --- | --- | --- | --- | --- | --- | --- | --- | --- | --- | --- | --- | --- | --- |
|  | **fp2** | **fp3** | **fp4** | **maccs** | **fp2** | **fp3** | **fp4** | **maccs** | **fp2** | **fp3** | **fp4** | **maccs** | **fp2** | **fp3** | **fp4** | **maccs** |
| **CFX** | | | | | | | | | | | | | | | | |
| **p=1** | -0.27 (0.06) | -0.04 (0.06) | -0.11 (0.05) | -0.08 (0.06) | -0.35  (0.06) | -0.12 (0.05) | -0.19 (0.05) | -0.15 (0.06) | 0.14 (0.01) | 0.00 (0.00) | 0.04 (0.01) | 0.02 (0.00) | 0.15 (0.02) | 0.00 (0.00) | 0.05 (0.01) | 0.03 (0.01) |
| **p=2** | -0.37  (0.06) | -0.04 (0.06) | -0.16 (0.05) | -0.11 (0.06) | -0.46 (0.06) | -0.12 (0.06) | -0.24 (0.05) | -0.19 (0.06) | 0.20 (0.02) | 0.00 (0.00) | 0.07 (0.01) | 0.05 (0.01) | 0.20 (0.03) | 0.00 (0.00) | 0.08 (0.01) | 0.05 (0.01) |
| **p=3** | -0.49  (0.06) | -0.04 (0.06) | -0.21 (0.05) | -0.16 (0.06) | -0.58 (0.06) | -0.12 (0.06) | -0.30 (0.05) | -0.24 (0.06) | 0.27 (0.02) | 0.00 (0.00) | 0.11 (0.01) | 0.07 (0.01) | 0.25 (0.03) | 0.00 (0.00) | 0.11 (0.02) | 0.08 (0.01) |
| **p=4** | -0.60  (0.06) | -0.04 (0.06) | -0.29 (0.06) | -0.21 (0.06) | -0.71 (0.06) | -0.12 (0.06) | -0.38 (0.05) | -0.30 (0.06) | 0.34 (0.02) | 0.00 (0.00) | 0.15 (0.01) | 0.11 (0.01) | 0.30 (0.04) | 0.00 (0.00) | 0.15 (0.02) | 0.11 (0.02) |
| **D2R** | | | | | | | | | | | | | | | | |
| **p=1** | -0.66 (0.07) | -0.57 (0.08) | -0.58 (0.08) | -0.58 (0.08) | -0.70 (0.07) | -0.59 (0.07) | -0.61 (0.07) | -0.61 (0.07) | 0.02 (0.01) | 0.00 (0.00) | 0.00 (0.00) | 0.00 (0.00) | 0.09 (0.01) | -0.01 (0.00) | 0.01 (0.00) | 0.01 (0.00) |
| **p=2** | -0.72 (0.08) | -0.57 (0.08) | -0.60 (0.08) | -0.59 (0.08) | -0.78 (0.07) | -0.59 (0.07) | -0.63 (0.07) | -0.63 (0.07) | 0.05 (0.01) | 0.00 (0.00) | 0.00 (0.00) | 0.00 (0.00) | 0.15 (0.01) | -0.01 (0.00) | 0.02 (0.00) | 0.02 (0.00) |
| **p=3** | -0.81 (0.08) | -0.57 (0.07) | -0.62 (0.08) | -0.61 (0.08) | -0.88 (0.06) | -0.60 (0.07) | -0.66 (0.07) | -0.64 (0.07) | 0.09 (0.02) | 0.00 (0.00) | 0.01 (0.00) | 0.01 (0.00) | 0.23 (0.02) | -0.01 (0.01) | 0.05 (0.01) | 0.03 (0.00) |
| **p=4** | -0.89 (0.08) | -0.57 (0.07) | -0.65 (0.08) | -0.63 (0.08) | -0.99 (0.06) | -0.60 (0.07) | -0.69 (0.07) | -0.67 (0.07) | 0.16 (0.03) | 0.00 (0.00) | 0.02 (0.01) | 0.01 (0.00) | 0.32 (0.02) | -0.01 (0.01) | 0.08 (0.01) | 0.05 (0.00) |
| **ERK2** | | | | | | | | | | | | | | | | |
| **p=1** | -0.22 (0.06) | -0.11 (0.06) | -0.14 (0.06) | -0.12 (0.06) | -0.29 (0.07) | -0.17 (0.06) | -0.20 (0.06) | -0.19 (0.06) | 0.15 (0.02) | 0.00 (0.00) | 0.02 (0.00) | 0.02 (0.00) | 0.04 (0.01) | 0.00 (0.00) | 0.01 (0.00) | 0.01 (0.00) |
| **p=2** | -0.25 (0.06) | -0.11 (0.06) | -0.17 (0.06) | -0.14 (0.06) | -0.33 (0.07) | -0.18 (0.06) | -0.24 (0.06) | -0.21 (0.06) | 0.18 (0.02) | 0.01 (0.00) | 0.05 (0.00) | 0.03 (0.00) | 0.05 (0.01) | 0.00 (0.00) | 0.02 (0.00) | 0.01 (0.00) |
| **p=3** | -0.29 (0.06) | -0.12 (0.07) | -0.20 (0.06) | -0.16 (0.06) | -0.37 (0.07) | -0.18 (0.06) | -0.28 (0.06) | -0.23 (0.06) | 0.20 (0.02) | 0.01 (0.00) | 0.09 (0.01) | 0.06 (0.01) | 0.06 (0.01) | 0.00 (0.00) | 0.03 (0.00) | 0.02 (0.00) |
| **p=4** | -0.32 (0.06) | -0.13 (0.07) | -0.24 (0.06) | -0.18 (0.06) | -0.40 (0.08) | -0.19 (0.07) | -0.32 (0.06) | -0.26 (0.07) | 0.22 (0.02) | 0.02 (0.01) | 0.13 (0.01) | 0.09 (0.01) | 0.07 (0.01) | 0.01 (0.00) | 0.04 (0.01) | 0.03 (0.01) |
| **ER** | | | | | | | | | | | | | | | | |
| **p=1** | -0.47 (0.11) | -0.11 (0.09) | -0.18 (0.10) | -0.44 (0.10) | -0.62 (0.13) | -0.23 (0.08) | -0.32 (0.10) | -0.59 (0.11) | 0.23 (0.06) | 0.03 (0.01) | 0.09 (0.02) | 0.22 (0.04) | 0.14 (0.05) | 0.02 (0.02) | 0.06 (0.03) | 0.14 (0.04) |
| **p=2** | -0.59 (0.12) | -0.20 (0.08) | -0.33 (0.11) | -0.56 (0.11) | -0.74 (0.15) | -0.32 (0.09) | -0.48 (0.11) | -0.71 (0.13) | 0.28 (0.06) | 0.08 (0.02) | 0.18 (0.04) | 0.27 (0.05) | 0.16 (0.05) | 0.05 (0.03) | 0.13 (0.04) | 0.16 (0.04) |
| **p=3** | -0.69 (0.12) | -0.27 (0.09) | -0.46 (0.10) | -0.64 (0.12) | -0.83 (0.16) | -0.39 (0.09) | -0.62 (0.12) | -0.79 (0.14) | 0.32 (0.06) | 0.12 (0.03) | 0.24 (0.04) | 0.30 (0.06) | 0.18 (0.05) | 0.06 (0.03) | 0.15 (0.04) | 0.18 (0.05) |
| **p=4** | -0.73 (0.13) | -0.32 (0.09) | -0.56 (0.09) | -0.71 (0.12) | -0.87 (0.17) | -0.43 (0.10) | -0.71 (0.12) | -0.85 (0.16) | 0.33 (0.06) | 0.15 (0.04) | 0.27 (0.05) | 0.33 (0.06) | 0.19 (0.05) | 0.07 (0.04) | 0.17 (0.04) | 0.19 (0.05) |
| **MOR** | | | | | | | | | | | | | | | | |
| **p=1** | -1.31 (0.26) | -1.11 (0.28) | -1.17 (0.29) | -1.11 (0.30) | -1.47 (0.21) | -1.27 (0.23) | -1.32 (0.23) | -1.25 (0.24) | 0.09 (0.03) | 0.00 (0.00) | 0.02 (0.01) | 0.00 (0.00) | 0.21 (0.04) | 0.01 (0.01) | 0.07 (0.02) | 0.02 (0.01) |
| **p=2** | -1.34 (0.25) | -1.13 (0.28) | -1.21 (0.29) | -1.14 (0.30) | -1.51 (0.21) | -1.28 (0.23) | -1.36 (0.23) | -1.28 (0.24) | 0.11 (0.03) | 0.01 (0.01) | 0.03 (0.01) | 0.01 (0.01) | 0.24 (0.04) | 0.02 (0.01) | 0.11 (0.02) | 0.05 (0.01) |
| **p=3** | -1.37 (0.24) | -1.14 (0.28) | -1.26 (0.28) | -1.18 (0.30) | -1.55 (0.20) | -1.30 (0.23) | -1.41 (0.23) | -1.32 (0.24) | 0.13 (0.03) | 0.01 (0.01) | 0.05 (0.02) | 0.02 (0.01) | 0.27 (0.05) | 0.04 (0.01) | 0.15 (0.03) | 0.09 (0.01) |
| **p=4** | -1.41 (0.23) | -1.16 (0.28) | -1.31 (0.28) | -1.22 (0.30) | -1.61 (0.19) | -1.31 (0.23) | -1.46 (0.22) | -1.36 (0.24) | 0.16 (0.04) | 0.02 (0.01) | 0.07 (0.02) | 0.04 (0.02) | 0.30 (0.05) | 0.05 (0.02) | 0.19 (0.03) | 0.13 (0.02) |
| **VEGFR2** | | | | | | | | | | | | | | | | |
| **p=1** | 0.04 (0.08) | 0.10 (0.08) | 0.10 (0.08) | 0.09 (0.08) | -0.11 (0.08) | -0.02 (0.08) | -0.03 (0.08) | -0.04 (0.08) | 0.05 (0.01) | 0.00 (0.00) | 0.01 (0.00) | 0.01 (0.00) | 0.05 (0.01) | 0.00 (0.00) | 0.01 (0.00) | 0.01 (0.00) |
| **p=2** | 0.00 (0.08) | 0.10 (0.08) | 0.09 (0.08) | 0.07 (0.08) | -0.16 (0.08) | -0.03 (0.08) | -0.05 (0.08) | -0.07 (0.08) | 0.08 (0.01) | 0.01 (0.00) | 0.02 (0.00) | 0.02 (0.00) | 0.07 (0.01) | 0.01 (0.00) | 0.02 (0.00) | 0.02 (0.01) |
| **p=3** | -0.04 (0.08) | 0.09 (0.08) | 0.07 (0.08) | 0.04 (0.08) | -0.21 (0.08) | -0.04 (0.08) | -0.07 (0.09) | -0.09 (0.08) | 0.11 (0.02) | 0.01 (0.00) | 0.03 (0.00) | 0.03 (0.01) | 0.10 (0.01) | 0.02 (0.00) | 0.03 (0.01) | 0.04 (0.01) |
| **p=4** | -0.09 (0.08) | 0.08 (0.08) | 0.04 (0.09) | 0.02 (0.08) | -0.25 (0.09) | -0.05 (0.08) | -0.11 (0.09) | -0.13 (0.08) | 0.14 (0.02) | 0.02 (0.00) | 0.05 (0.01) | 0.05 (0.01) | 0.13 (0.02) | 0.02 (0.00) | 0.05 (0.01) | 0.05 (0.01) |
| **5HT2AR** | | | | | | | | | | | | | | | | |
| **p=1** | -0.85 (0.07) | -0.65 (0.07) | -0.67 (0.07) | -0.67 (0.07) | -0.98 (0.08) | -0.80 (0.07) | -0.83 (0.07) | -0.82 (0.07) | 0.13 (0.02) | 0.00 (0.00) | 0.01 (0.00) | 0.01 (0.00) | 0.17 (0.01) | 0.00 (0.00) | 0.03 (0.00) | 0.01 (0.00) |
| **p=2** | -0.93 (0.07) | -0.66 (0.07) | -0.71 (0.07) | -0.69 (0.07) | -1.07 (0.07) | -0.80 (0.07) | -0.87 (0.07) | -0.84 (0.07) | 0.19 (0.02) | 0.00 (0.00) | 0.03 (0.00) | 0.02 (0.00) | 0.24 (0.02) | 0.00 (0.00) | 0.07 (0.01) | 0.03 (0.00) |
| **p=3** | -1.01 (0.07) | -0.66 (0.07) | -0.76 (0.08) | -0.72 (0.07) | -1.18 (0.07) | -0.80 (0.07) | -0.92 (0.07) | -0.87 (0.07) | 0.26 (0.02) | 0.00 (0.00) | 0.06 (0.01) | 0.03 (0.00) | 0.33 (0.02) | 0.01 (0.00) | 0.12 (0.01) | 0.06 (0.00) |
| **p=4** | -1.10 (0.07) | -0.66 (0.07) | -0.82 (0.07) | -0.75 (0.07) | -1.27 (0.06) | -0.81 (0.08) | -0.98 (0.07) | -0.91 (0.07) | 0.34 (0.02) | 0.01 (0.00) | 0.10 (0.01) | 0.05 (0.01) | 0.40 (0.03) | 0.01 (0.00) | 0.17 (0.01) | 0.09 (0.01) |
| **A2AR** | | | | | | | | | | | | | | | | |
| **p=1** | -0.36 (0.05) | -0.02 (0.03) | -0.10 (0.04) | -0.04 (0.03) | -0.35 (0.06) | -0.01 (0.04) | -0.11 (0.05) | -0.04 (0.03) | 0.23 (0.02) | 0.00 (0.00) | 0.05 (0.01) | 0.01 (0.00) | 0.27 (0.02) | 0.00 (0.00) | 0.08 (0.01) | 0.02 (0.00) |
| **p=2** | -0.43 (0.05) | -0.03 (0.03) | -0.15 (0.04) | -0.06 (0.03) | -0.43 (0.06) | -0.02 (0.04) | -0.15 (0.05) | -0.06 (0.04) | 0.29 (0.02) | 0.01 (0.00) | 0.08 (0.02) | 0.02 (0.00) | 0.32 (0.03) | 0.02 (0.00) | 0.11 (0.02) | 0.04 (0.00) |
| **p=3** | -0.51 (0.05) | -0.05 (0.03) | -0.20 (0.04) | -0.09 (0.03) | -0.51 (0.06) | -0.04 (0.04) | -0.21 (0.05) | -0.08 (0.04) | 0.34 (0.02) | 0.02 (0.00) | 0.12 (0.02) | 0.04 (0.00) | 0.36 (0.03) | 0.03 (0.01) | 0.16 (0.02) | 0.06 (0.01) |
| **p=4** | -0.56 (0.06) | -0.06 (0.03) | -0.26 (0.04) | -0.13 (0.03) | -0.58 (0.07) | -0.06 (0.04) | -0.27 (0.05) | -0.12 (0.04) | 0.38 (0.02) | 0.03 (0.01) | 0.16 (0.02) | 0.06 (0.01) | 0.40 (0.03) | 0.04 (0.01) | 0.20 (0.02) | 0.09 (0.01) |
| **CB1** | | | | | | | | | | | | | | | | |
| **p=1** | -0.40 (0.06) | -0.04 (0.01) | -0.11 (0.02) | -0.08 (0.02) | -0.40 (0.08) | -0.05 (0.01) | -0.12 (0.01) | -0.09 (0.01) | 0.22 (0.04) | 0.00 (0.00) | 0.03 (0.01) | 0.01 (0.00) | 0.34 (0.06) | 0.00 (0.00) | 0.06 (0.01) | 0.03 (0.01) |
| **p=2** | -0.44 (0.07) | -0.05 (0.01) | -0.16 (0.02) | -0.13 (0.02) | -0.46 (0.09) | -0.05 (0.01) | -0.17 (0.02) | -0.15 (0.01) | 0.27 (0.04) | 0.00 (0.00) | 0.05 (0.01) | 0.04 (0.01) | 0.38 (0.06) | 0.01 (0.01) | 0.12 (0.02) | 0.09 (0.02) |
| **p=3** | -0.49 (0.07) | -0.06 (0.02) | -0.22 (0.02) | -0.19 (0.02) | -0.51 (0.10) | -0.06 (0.01) | -0.23 (0.03) | -0.21 (0.02) | 0.31 (0.05) | 0.01 (0.00) | 0.09 (0.01) | 0.07 (0.01) | 0.42 (0.07) | 0.02 (0.01) | 0.18 (0.02) | 0.15 (0.02) |
| **p=4** | -0.53 (0.08) | -0.06 (0.02) | -0.27 (0.03) | -0.25 (0.02) | -0.56 (0.10) | -0.06 (0.02) | -0.29 (0.04) | -0.28 (0.02) | 0.35 (0.05) | 0.01 (0.01) | 0.13 (0.02) | 0.11 (0.01) | 0.44 (0.07) | 0.03 (0.02) | 0.24 (0.03) | 0.22 (0.03) |
| **M1R** | | | | | | | | | | | | | | | | |
| **p=1** | -0.26 (0.06) | 0.04 (0.05) | 0.01 (0.07) | 0.01 (0.05) | -0.36 (0.07) | -0.05 (0.05) | -0.09 (0.06) | -0.08 (0.05) | 0.23 (0.03) | 0.01 (0.00) | 0.03 (0.01) | 0.02 (0.00) | 0.27 (0.04) | 0.01 (0.01) | 0.05 (0.01) | 0.03 (0.01) |
| **p=2** | -0.35 (0.05) | 0.02 (0.05) | -0.06 (0.07) | -0.03 (0.05) | -0.45 (0.07) | -0.07 (0.05) | -0.17 (0.06) | -0.12 (0.05) | 0.28 (0.03) | 0.02 (0.01) | 0.08 (0.02) | 0.05 (0.01) | 0.33 (0.05) | 0.02 (0.01) | 0.10 (0.01) | 0.06 (0.01) |
| **p=3** | -0.42 (0.05) | 0.00 (0.06) | -0.14 (0.07) | -0.09 (0.05) | -0.51 (0.07) | -0.10 (0.05) | -0.26 (0.06) | -0.19 (0.05) | 0.32 (0.03) | 0.03 (0.01) | 0.14 (0.02) | 0.09 (0.01) | 0.36 (0.05) | 0.05 (0.01) | 0.18 (0.01) | 0.11 (0.01) |
| **p=4** | -0.47 (0.05) | -0.02 (0.06) | -0.23 (0.07) | -0.16 (0.06) | -0.55 (0.07) | -0.12 (0.06) | -0.35 (0.06) | -0.27 (0.05) | 0.35 (0.03) | 0.05 (0.01) | 0.20 (0.02) | 0.14 (0.02) | 0.38 (0.05) | 0.07 (0.01) | 0.24 (0.02) | 0.17 (0.02) |
| **rRNA** | | | | | | | | | | | | | | | | |
| **p=1** | -0.99 (0.21) | -0.65 (0.21) | -0.81 (0.20) | -0.76 (0.20) | -1.32 (0.19) | -0.92 (0.16) | -1.12 (0.15) | -1.04 (0.16) | 0.38 (0.11) | 0.06 (0.02) | 0.21 (0.08) | 0.14 (0.06) | 0.51 (0.15) | 0.14 (0.04) | 0.35 (0.11) | 0.32 (0.13) |
| **p=2** | -1.07 (0.22) | -0.75 (0.22) | -0.91 (0.20) | -0.88 (0.19) | -1.41 (0.20) | -1.02 (0.17) | -1.23 (0.16) | -1.19 (0.16) | 0.44 (0.12) | 0.14 (0.04) | 0.29 (0.10) | 0.27 (0.09) | 0.53 (0.16) | 0.30 (0.09) | 0.44 (0.13) | 0.45 (0.15) |
| **p=3** | -1.12 (0.23) | -0.82 (0.23) | -0.98 (0.20) | -0.98 (0.19) | -1.46 (0.21) | -1.11 (0.18) | -1.31 (0.18) | -1.30 (0.17) | 0.48 (0.12) | 0.22 (0.06) | 0.35 (0.12) | 0.37 (0.12) | 0.53 (0.14) | 0.40 (0.11) | 0.46 (0.14) | 0.50 (0.15) |
| **p=4** | -1.15 (0.23) | -0.88 (0.25) | -1.02 (0.20) | -1.05 (0.20) | -1.49 (0.22) | -1.16 (0.20) | -1.36 (0.19) | -1.39 (0.19) | 0.50 (0.13) | 0.27 (0.07) | 0.39 (0.14) | 0.44 (0.13) | 0.54 (0.14) | 0.46 (0.11) | 0.48 (0.15) | 0.51 (0.15) |

**Table S5.** Mean RMSE (kcal/mol), MAE (kcal/mol), R^2^ and PI values before and after calibration of AutoDock Vina docking scores for all receptors under the conditions of different fingerprint type and *p* value in CSE function (*S^p^*). “*orig*” refers to original, representing the initial situation before the calibration. 95% CI for each metrics is displayed in the parenthesis.

| **Function** | **MAE** | | | | **RMSE** | | | | **R^2^** | | | | **PI** | | | |
| --- | --- | --- | --- | --- | --- | --- | --- | --- | --- | --- | --- | --- | --- | --- | --- | --- |
|  | **fp2** | **fp3** | **fp4** | **maccs** | **fp2** | **fp3** | **fp4** | **maccs** | **fp2** | **fp3** | **fp4** | **maccs** | **fp2** | **fp3** | **fp4** | **maccs** |
| **CFX (Tc > 0.35)** | | | | | | | | | | | | | | | | |
| **orig** | 1.96 (0.07) | | | | 2.35 (0.09) | | | | 0.12 (0.03) | | | | 0.33 (0.05) | | | |
| **p=1** | 1.72 (0.06) | 1.96 (0.07) | 1.91 (0.07) | 1.93 (0.07) | 2.09 (0.08) | 2.35 (0.09) | 2.29 (0.08) | 2.31 (0.09) | 0.30 (0.04) | 0.12 (0.03) | 0.16 (0.03) | 0.14 (0.03) | 0.57 (0.04) | 0.32 (0.05) | 0.39 (0.04) | 0.36 (0.05) |
| **p=2** | 1.62 (0.05) | 1.96 (0.07) | 1.88 (0.06) | 1.91 (0.07) | 1.98 (0.07) | 2.34 (0.09) | 2.25 (0.08) | 2.29 (0.09) | 0.37 (0.04) | 0.12 (0.03) | 0.18 (0.03) | 0.16 (0.03) | 0.62 (0.03) | 0.33 (0.05) | 0.43 (0.04) | 0.39 (0.04) |
| **p=3** | 1.51 (0.04) | 1.96 (0.07) | 1.83 (0.06) | 1.88 (0.06) | 1.86 (0.07) | 2.34 (0.09) | 2.21 (0.08) | 2.25 (0.08) | 0.44 (0.04) | 0.12 (0.03) | 0.22 (0.03) | 0.18 (0.03) | 0.68 (0.03) | 0.34 (0.05) | 0.47 (0.03) | 0.43 (0.04) |
| **p=4** | 1.40 (0.04) | 1.95 (0.07) | 1.78 (0.06) | 1.84 (0.06) | 1.76 (0.07) | 2.33 (0.09) | 2.14 (0.07) | 2.21 (0.08) | 0.50 (0.04) | 0.13 (0.03) | 0.26 (0.03) | 0.21 (0.03) | 0.72 (0.03) | 0.34 (0.05) | 0.52 (0.03) | 0.47 (0.04) |
| **D2R (Tc > 0.35)** | | | | | | | | | | | | | | | | |
| **orig** | 1.85 (0.05) | | | | 2.23 (0.06) | | | | 0.03 (0.01) | | | | 0.14 (0.04) | | | |
| **p=1** | 1.53 (0.07) | 1.75 (0.06) | 1.74 (0.06) | 1.73 (0.06) | 1.90 (0.08) | 2.10 (0.06) | 2.09 (0.06) | 2.08 (0.06) | 0.13 (0.03) | 0.03 (0.01) | 0.03 (0.01) | 0.03 (0.01) | 0.37 (0.04) | 0.14 (0.04) | 0.15 (0.04) | 0.16 (0.04) |
| **p=2** | 1.48 (0.07) | 1.74 (0.06) | 1.71 (0.06) | 1.71 (0.06) | 1.84 (0.07) | 2.09 (0.06) | 2.07 (0.06) | 2.06 (0.06) | 0.16 (0.04) | 0.03 (0.01) | 0.03 (0.01) | 0.03 (0.01) | 0.42 (0.04) | 0.15 (0.04) | 0.17 (0.04) | 0.17 (0.04) |
| **p=3** | 1.41 (0.07) | 1.73 (0.06) | 1.68 (0.06) | 1.69 (0.06) | 1.77 (0.07) | 2.08 (0.06) | 2.04 (0.06) | 2.04 (0.06) | 0.21 (0.04) | 0.03 (0.01) | 0.04 (0.01) | 0.04 (0.02) | 0.47 (0.04) | 0.16 (0.04) | 0.20 (0.04) | 0.19 (0.04) |
| **p=4** | 1.35 (0.06) | 1.72 (0.06) | 1.64 (0.06) | 1.66 (0.06) | 1.71 (0.07) | 2.06 (0.06) | 2.00 (0.06) | 2.01 (0.06) | 0.26 (0.04) | 0.03 (0.01) | 0.05 (0.02) | 0.05 (0.02) | 0.52 (0.04) | 0.17 (0.04) | 0.23 (0.04) | 0.21 (0.04) |
| **ERK2 (Tc > 0.30)** | | | | | | | | | | | | | | | | |
| **orig** | 1.48 (0.05) | | | | 1.78 (0.05) | | | | 0.11 (0.02) | | | | 0.54 (0.04) | | | |
| **p=1** | 0.87 (0.03) | 1.14 (0.03) | 1.07 (0.03) | 1.11 (0.03) | 1.10 (0.05) | 1.52 (0.05) | 1.42 (0.04) | 1.49 (0.04) | 0.53 (0.04) | 0.12 (0.01) | 0.21 (0.02) | 0.15 (0.02) | 0.78 (0.02) | 0.55 (0.03) | 0.68 (0.03) | 0.61 (0.03) |
| **p=2** | 0.80 (0.03) | 1.12 (0.03) | 1.02 (0.03) | 1.08 (0.03) | 1.03 (0.05) | 1.48 (0.05) | 1.32 (0.04) | 1.42 (0.04) | 0.60 (0.04) | 0.16 (0.02) | 0.31 (0.03) | 0.21 (0.02) | 0.82 (0.02) | 0.60 (0.03) | 0.73 (0.03) | 0.68 (0.02) |
| **p=3** | 0.73 (0.03) | 1.10 (0.03) | 0.95 (0.03) | 1.03 (0.03) | 0.95 (0.05) | 1.44 (0.04) | 1.21 (0.04) | 1.34 (0.04) | 0.65 (0.04) | 0.20 (0.02) | 0.42 (0.03) | 0.29 (0.02) | 0.85 (0.02) | 0.65 (0.02) | 0.76 (0.03) | 0.73 (0.02) |
| **p=4** | 0.67 (0.03) | 1.07 (0.03) | 0.88 (0.03) | 0.97 (0.02) | 0.89 (0.05) | 1.39 (0.04) | 1.10 (0.04) | 1.24 (0.03) | 0.70 (0.04) | 0.24 (0.02) | 0.52 (0.04) | 0.39 (0.03) | 0.87 (0.02) | 0.68 (0.02) | 0.79 (0.03) | 0.76 (0.03) |
| **ER (Tc > 0.35)** | | | | | | | | | | | | | | | | |
| **orig** | 1.76 (0.11) | | | | 2.17 (0.12) | | | | 0.14 (0.04) | | | | 0.36 (0.07) | | | |
| **p=1** | 1.32 (0.13) | 1.73 (0.08) | 1.56 (0.08) | 1.44 (0.08) | 1.70 (0.17) | 2.15 (0.09) | 1.92 (0.10) | 1.86 (0.09) | 0.49 (0.09) | 0.16 (0.05) | 0.31 (0.05) | 0.37 (0.05) | 0.69 (0.07) | 0.39 (0.07) | 0.57 (0.05) | 0.60 (0.04) |
| **p=2** | 1.30 (0.14) | 1.71 (0.08) | 1.48 (0.07) | 1.38 (0.08) | 1.68 (0.19) | 2.13 (0.09) | 1.83 (0.09) | 1.80 (0.09) | 0.50 (0.09) | 0.18 (0.05) | 0.38 (0.05) | 0.40 (0.05) | 0.70 (0.07) | 0.42 (0.07) | 0.62 (0.04) | 0.63 (0.04) |
| **p=3** | 1.30 (0.14) | 1.69 (0.08) | 1.40 (0.08) | 1.32 (0.09) | 1.69 (0.20) | 2.12 (0.09) | 1.75 (0.09) | 1.74 (0.09) | 0.51 (0.10) | 0.19 (0.05) | 0.43 (0.05) | 0.44 (0.05) | 0.71 (0.08) | 0.43 (0.07) | 0.66 (0.04) | 0.66 (0.04) |
| **p=4** | 1.30 (0.14) | 1.69 (0.08) | 1.34 (0.09) | 1.25 (0.09) | 1.70 (0.21) | 2.11 (0.08) | 1.70 (0.10) | 1.68 (0.10) | 0.51 (0.10) | 0.20 (0.05) | 0.47 (0.05) | 0.48 (0.05) | 0.71 (0.08) | 0.44 (0.07) | 0.69 (0.04) | 0.68 (0.04) |
| **MOR (Tc > 0.30)** | | | | | | | | | | | | | | | | |
| **orig** | 1.73 (0.05) | | | | 2.10 (0.05) | | | | 0.10 (0.03) | | | | 0.31 (0.06) | | | |
| **p=1** | 1.60 (0.06) | 1.73 (0.05) | 1.71 (0.05) | 1.74 (0.05) | 1.95 (0.05) | 2.08 (0.05) | 2.06 (0.05) | 2.08 (0.04) | 0.24 (0.04) | 0.11 (0.04) | 0.14 (0.04) | 0.11 (0.04) | 0.49 (0.04) | 0.34 (0.06) | 0.37 (0.06) | 0.33 (0.06) |
| **p=2** | 1.55 (0.06) | 1.71 (0.05) | 1.67 (0.05) | 1.71 (0.05) | 1.90 (0.05) | 2.06 (0.05) | 2.01 (0.04) | 2.05 (0.04) | 0.28 (0.04) | 0.13 (0.04) | 0.17 (0.04) | 0.14 (0.04) | 0.54 (0.04) | 0.36 (0.06) | 0.42 (0.05) | 0.37 (0.06) |
| **p=3** | 1.49 (0.06) | 1.69 (0.05) | 1.62 (0.05) | 1.69 (0.05) | 1.84 (0.05) | 2.04 (0.05) | 1.96 (0.04) | 2.02 (0.04) | 0.33 (0.03) | 0.15 (0.04) | 0.22 (0.04) | 0.17 (0.04) | 0.59 (0.03) | 0.38 (0.06) | 0.48 (0.05) | 0.41 (0.05) |
| **p=4** | 1.42 (0.05) | 1.67 (0.05) | 1.57 (0.05) | 1.65 (0.05) | 1.78 (0.06) | 2.02 (0.05) | 1.90 (0.04) | 1.97 (0.04) | 0.38 (0.03) | 0.16 (0.05) | 0.27 (0.04) | 0.20 (0.04) | 0.64 (0.03) | 0.40 (0.07) | 0.53 (0.04) | 0.45 (0.05) |
| **VEGFR2 (Tc > 0.25)** | | | | | | | | | | | | | | | | |
| **orig** | 1.43 (0.04) | | | | 1.80 (0.06) | | | | 0.42 (0.04) | | | | 0.66 (0.03) | | | |
| **p=1** | 1.24 (0.03) | 1.36 (0.03) | 1.34 (0.03) | 1.34 (0.03) | 1.59 (0.05) | 1.74 (0.05) | 1.71 (0.05) | 1.72 (0.05) | 0.51 (0.04) | 0.42 (0.04) | 0.44 (0.04) | 0.43 (0.04) | 0.72 (0.02) | 0.66 (0.03) | 0.67 (0.02) | 0.67 (0.03) |
| **p=2** | 1.18 (0.03) | 1.35 (0.03) | 1.31 (0.03) | 1.32 (0.03) | 1.50 (0.04) | 1.72 (0.05) | 1.68 (0.05) | 1.69 (0.05) | 0.56 (0.03) | 0.43 (0.04) | 0.46 (0.04) | 0.45 (0.04) | 0.76 (0.02) | 0.67 (0.03) | 0.69 (0.02) | 0.68 (0.03) |
| **p=3** | 1.10 (0.03) | 1.34 (0.03) | 1.28 (0.03) | 1.30 (0.03) | 1.41 (0.03) | 1.72 (0.05) | 1.65 (0.06) | 1.66 (0.05) | 0.62 (0.03) | 0.43 (0.04) | 0.48 (0.04) | 0.47 (0.04) | 0.80 (0.02) | 0.67 (0.03) | 0.70 (0.02) | 0.70 (0.03) |
| **p=4** | 1.02 (0.02) | 1.33 (0.03) | 1.24 (0.03) | 1.27 (0.03) | 1.32 (0.03) | 1.71 (0.05) | 1.61 (0.06) | 1.62 (0.06) | 0.67 (0.02) | 0.44 (0.04) | 0.50 (0.04) | 0.49 (0.04) | 0.83 (0.01) | 0.68 (0.03) | 0.72 (0.02) | 0.71 (0.03) |
| **5HT2AR (Tc > 0.20)** | | | | | | | | | | | | | | | | |
| **orig** | 1.59 (0.09) | | | | 1.98 (0.09) | | | | 0.11 (0.03) | | | | 0.36 (0.05) | | | |
| **p=1** | 1.54 (0.08) | 1.60 (0.09) | 1.57 (0.08) | 1.57 (0.08) | 1.92 (0.09) | 1.99 (0.09) | 1.95 (0.09) | 1.95 (0.08) | 0.13 (0.03) | 0.11 (0.03) | 0.11 (0.03) | 0.11 (0.03) | 0.39 (0.05) | 0.35 (0.05) | 0.36 (0.05) | 0.37 (0.05) |
| **p=2** | 1.48 (0.08) | 1.60 (0.09) | 1.54 (0.08) | 1.55 (0.08) | 1.85 (0.09) | 1.99 (0.09) | 1.92 (0.08) | 1.92 (0.08) | 0.17 (0.04) | 0.11 (0.03) | 0.13 (0.03) | 0.12 (0.03) | 0.43 (0.05) | 0.35 (0.05) | 0.38 (0.05) | 0.38 (0.05) |
| **p=3** | 1.39 (0.08) | 1.60 (0.09) | 1.50 (0.08) | 1.52 (0.08) | 1.75 (0.09) | 1.99 (0.09) | 1.88 (0.08) | 1.89 (0.08) | 0.23 (0.04) | 0.11 (0.03) | 0.15 (0.04) | 0.14 (0.03) | 0.50 (0.05) | 0.35 (0.05) | 0.41 (0.05) | 0.40 (0.05) |
| **p=4** | 1.27 (0.07) | 1.60 (0.09) | 1.45 (0.08) | 1.48 (0.08) | 1.62 (0.09) | 2.00 (0.09) | 1.82 (0.08) | 1.85 (0.08) | 0.32 (0.05) | 0.11 (0.03) | 0.18 (0.04) | 0.16 (0.04) | 0.58 (0.04) | 0.35 (0.05) | 0.44 (0.04) | 0.42 (0.05) |
| **A2AR (Tc > 0.35)** | | | | | | | | | | | | | | | | |
| **orig** | 1.89 (0.05) | | | | 2.33 (0.06) | | | | 0.03 (0.02) | | | | 0.14 (0.05) | | | |
| **p=1** | 1.54 (0.06) | 1.90 (0.05) | 1.81 (0.05) | 1.87 (0.05) | 1.97 (0.09) | 2.34 (0.05) | 2.24 (0.05) | 2.31 (0.06) | 0.27 (0.05) | 0.03 (0.02) | 0.07 (0.02) | 0.04 (0.02) | 0.52 (0.05) | 0.15 (0.04) | 0.26 (0.04) | 0.18 (0.05) |
| **p=2** | 1.45 (0.06) | 1.88 (0.05) | 1.76 (0.05) | 1.85 (0.05) | 1.87 (0.09) | 2.32 (0.05) | 2.19 (0.05) | 2.28 (0.06) | 0.33 (0.06) | 0.04 (0.02) | 0.10 (0.03) | 0.05 (0.02) | 0.58 (0.05) | 0.18 (0.04) | 0.32 (0.04) | 0.20 (0.05) |
| **p=3** | 1.37 (0.06) | 1.86 (0.05) | 1.70 (0.04) | 1.82 (0.05) | 1.78 (0.09) | 2.31 (0.05) | 2.12 (0.05) | 2.25 (0.06) | 0.39 (0.06) | 0.04 (0.02) | 0.14 (0.03) | 0.06 (0.03) | 0.63 (0.05) | 0.20 (0.05) | 0.38 (0.04) | 0.24 (0.05) |
| **p=4** | 1.30 (0.06) | 1.84 (0.04) | 1.63 (0.04) | 1.78 (0.04) | 1.70 (0.10) | 2.29 (0.06) | 2.05 (0.05) | 2.20 (0.06) | 0.44 (0.06) | 0.05 (0.02) | 0.19 (0.03) | 0.09 (0.03) | 0.67 (0.04) | 0.22 (0.05) | 0.44 (0.04) | 0.28 (0.05) |
| **CB1 (Tc > 0.30)** | | | | | | | | | | | | | | | | |
| **orig** | 1.99 (0.11) | | | | 2.38 (0.12) | | | | 0.15 (0.04) | | | | 0.39 (0.06) | | | |
| **p=1** | 1.27 (0.06) | 1.45 (0.06) | 1.41 (0.06) | 1.45 (0.06) | 1.61 (0.08) | 1.80 (0.06) | 1.76 (0.06) | 1.81 (0.06) | 0.34 (0.05) | 0.17 (0.04) | 0.20 (0.04) | 0.17 (0.04) | 0.60 (0.04) | 0.41 (0.06) | 0.45 (0.05) | 0.41 (0.06) |
| **p=2** | 1.22 (0.06) | 1.44 (0.06) | 1.37 (0.06) | 1.42 (0.06) | 1.54 (0.08) | 1.79 (0.06) | 1.72 (0.06) | 1.77 (0.06) | 0.39 (0.05) | 0.17 (0.04) | 0.23 (0.04) | 0.19 (0.04) | 0.63 (0.04) | 0.42 (0.05) | 0.48 (0.05) | 0.45 (0.06) |
| **p=3** | 1.17 (0.07) | 1.43 (0.06) | 1.34 (0.06) | 1.38 (0.06) | 1.47 (0.08) | 1.78 (0.06) | 1.68 (0.06) | 1.73 (0.07) | 0.44 (0.05) | 0.18 (0.04) | 0.26 (0.05) | 0.23 (0.05) | 0.67 (0.03) | 0.43 (0.05) | 0.52 (0.04) | 0.49 (0.05) |
| **p=4** | 1.12 (0.07) | 1.42 (0.06) | 1.30 (0.06) | 1.33 (0.06) | 1.41 (0.08) | 1.77 (0.06) | 1.63 (0.07) | 1.68 (0.07) | 0.49 (0.05) | 0.19 (0.04) | 0.30 (0.05) | 0.27 (0.05) | 0.71 (0.03) | 0.44 (0.05) | 0.56 (0.04) | 0.53 (0.05) |
| **M1R (Tc > 0.30)** | | | | | | | | | | | | | | | | |
| **orig** | 1.62 (0.08) | | | | 1.99 (0.08) | | | | 0.31 (0.04) | | | | 0.54 (0.03) | | | |
| **p=1** | 1.33 (0.06) | 1.59 (0.08) | 1.54 (0.08) | 1.57 (0.08) | 1.65 (0.07) | 1.94 (0.08) | 1.88 (0.07) | 1.91 (0.08) | 0.49 (0.04) | 0.30 (0.04) | 0.34 (0.03) | 0.32 (0.04) | 0.71 (0.03) | 0.53 (0.04) | 0.57 (0.03) | 0.55 (0.03) |
| **p=2** | 1.27 (0.06) | 1.58 (0.08) | 1.50 (0.07) | 1.54 (0.08) | 1.59 (0.07) | 1.92 (0.08) | 1.83 (0.07) | 1.88 (0.08) | 0.52 (0.04) | 0.31 (0.04) | 0.37 (0.03) | 0.34 (0.04) | 0.73 (0.03) | 0.54 (0.04) | 0.61 (0.02) | 0.57 (0.03) |
| **p=3** | 1.22 (0.06) | 1.57 (0.08) | 1.45 (0.07) | 1.50 (0.08) | 1.53 (0.07) | 1.91 (0.08) | 1.76 (0.07) | 1.83 (0.08) | 0.56 (0.04) | 0.32 (0.04) | 0.41 (0.03) | 0.37 (0.04) | 0.75 (0.03) | 0.56 (0.04) | 0.65 (0.02) | 0.60 (0.03) |
| **p=4** | 1.18 (0.06) | 1.55 (0.08) | 1.39 (0.07) | 1.45 (0.08) | 1.48 (0.07) | 1.89 (0.08) | 1.69 (0.07) | 1.77 (0.08) | 0.58 (0.04) | 0.33 (0.04) | 0.46 (0.03) | 0.41 (0.04) | 0.77 (0.03) | 0.57 (0.03) | 0.69 (0.02) | 0.64 (0.03) |
| **rRNA (Tc > 0.30)** | | | | | | | | | | | | | | | | |
| **orig** | 1.88 (0.20) | | | | 2.10 (0.17) | | | | 0.26 (0.17) | | | | 0.48 (0.21) | | | |
| **p=1** | 0.64 (0.10) | 1.04 (0.07) | 0.70 (0.08) | 0.82 (0.07) | 0.79 (0.12) | 1.25 (0.09) | 0.91 (0.10) | 1.00 (0.10) | 0.71 (0.12) | 0.27 (0.16) | 0.63 (0.10) | 0.59 (0.13) | 0.77 (0.09) | 0.51 (0.22) | 0.79 (0.08) | 0.79 (0.08) |
| **p=2** | 0.63 (0.10) | 0.90 (0.07) | 0.67 (0.08) | 0.74 (0.08) | 0.79 (0.12) | 1.10 (0.09) | 0.85 (0.09) | 0.89 (0.09) | 0.71 (0.11) | 0.49 (0.17) | 0.68 (0.09) | 0.67 (0.12) | 0.79 (0.10) | 0.66 (0.16) | 0.80 (0.08) | 0.78 (0.07) |
| **p=3** | 0.63 (0.09) | 0.79 (0.08) | 0.65 (0.08) | 0.69 (0.08) | 0.80 (0.12) | 0.97 (0.10) | 0.82 (0.09) | 0.83 (0.09) | 0.71 (0.11) | 0.62 (0.14) | 0.70 (0.08) | 0.70 (0.11) | 0.78 (0.09) | 0.80 (0.06) | 0.80 (0.08) | 0.78 (0.07) |
| **p=4** | 0.63 (0.09) | 0.71 (0.09) | 0.64 (0.08) | 0.66 (0.08) | 0.80 (0.12) | 0.88 (0.09) | 0.81 (0.09) | 0.79 (0.10) | 0.70 (0.11) | 0.68 (0.12) | 0.70 (0.08) | 0.71 (0.11) | 0.78 (0.08) | 0.79 (0.08) | 0.80 (0.08) | 0.78 (0.07) |

**Table S6.** RMSE (kcal/mol), MAE (kcal/mol), R^2^ and PI values before and after calibration of the Glide docking scores for the external test sets of A2AR and CFX under the conditions of different fingerprint type and chemical structure effective function, *S^p^*, where *p* takes a value of 1, 2, 3 or 4. “orig” refers to original, representing the initial situation before the calibration.

| **Function** | **MAE** | | | | **RMSE** | | | | **R^2^** | | | | **PI** | | | |
| --- | --- | --- | --- | --- | --- | --- | --- | --- | --- | --- | --- | --- | --- | --- | --- | --- |
|  | **fp2** | **fp3** | **fp4** | **maccs** | **fp2** | **fp3** | **fp4** | **maccs** | **fp2** | **fp3** | **fp4** | **maccs** | **fp2** | **fp3** | **fp4** | **maccs** |
| **A2AR** | | | | | | | | | | | | | | | | |
| **orig** | 1.44 | | | | 1.78 | | | | 0.05 | | | | 0.24 | | | |
| **p=1** | 1.13 | 1.16 | 1.16 | 1.16 | 1.46 | 1.50 | 1.48 | 1.49 | 0.07 | 0.05 | 0.06 | 0.06 | 0.29 | 0.24 | 0.27 | 0.26 |
| **p=2** | 1.10 | 1.15 | 1.13 | 1.14 | 1.42 | 1.48 | 1.45 | 1.47 | 0.11 | 0.06 | 0.09 | 0.07 | 0.35 | 0.26 | 0.31 | 0.27 |
| **p=3** | 1.07 | 1.15 | 1.11 | 1.12 | 1.38 | 1.48 | 1.43 | 1.45 | 0.16 | 0.07 | 0.11 | 0.08 | 0.41 | 0.27 | 0.35 | 0.30 |
| **p=4** | 1.05 | 1.16 | 1.10 | 1.11 | 1.37 | 1.49 | 1.42 | 1.43 | 0.19 | 0.07 | 0.14 | 0.09 | 0.46 | 0.29 | 0.39 | 0.32 |
| **CFX** | | | | | | | | | | | | | | | | |
| **orig** | 2.71 | | | | 3.25 | | | | 0.02 | | | | 0.14 | | | |
| **p=1** | 2.02 | 2.07 | 2.03 | 2.03 | 2.54 | 2.60 | 2.55 | 2.54 | 0.03 | 0.02 | 0.03 | 0.03 | 0.19 | 0.14 | 0.17 | 0.17 |
| **p=2** | 1.93 | 2.07 | 1.99 | 1.98 | 2.43 | 2.59 | 2.50 | 2.49 | 0.06 | 0.02 | 0.04 | 0.04 | 0.25 | 0.14 | 0.21 | 0.20 |
| **p=3** | 1.80 | 2.07 | 1.93 | 1.94 | 2.29 | 2.59 | 2.44 | 2.44 | 0.10 | 0.02 | 0.05 | 0.05 | 0.33 | 0.15 | 0.24 | 0.22 |
| **p=4** | 1.66 | 2.07 | 1.87 | 1.89 | 2.13 | 2.59 | 2.37 | 2.39 | 0.16 | 0.02 | 0.07 | 0.06 | 0.42 | 0.15 | 0.28 | 0.26 |

**Table S7.** Mean AUC, EF_1%_ and EF_10%_ of screening results before and after calibration using the best hybrid scoring function (FP2 fingerprint with CSE=S^4^) for the external test sets of A2AR and CFX. “*cali*” and “*orig*” represent the calibrated and original docking scores, respectively. The number of compounds allocated in the active set and inactive set is shown in the table.

| Receptor | EF_10%_ | | EF_40%_ | | AUC | | Actives | Inactives |
| --- | --- | --- | --- | --- | --- | --- | --- | --- |
|  | *cali* | *orig* | *cali* | *orig* | *cali* | *orig* |  |  |
| CFX | 1.35 | 1.18 | 1.32 | 1.12 | 0.71 | 0.58 | 1182 | 417 |
| D2R | 1.06 | 0.98 | 1.28 | 1.13 | 0.71 | 0.61 | 1212 | 761 |

**
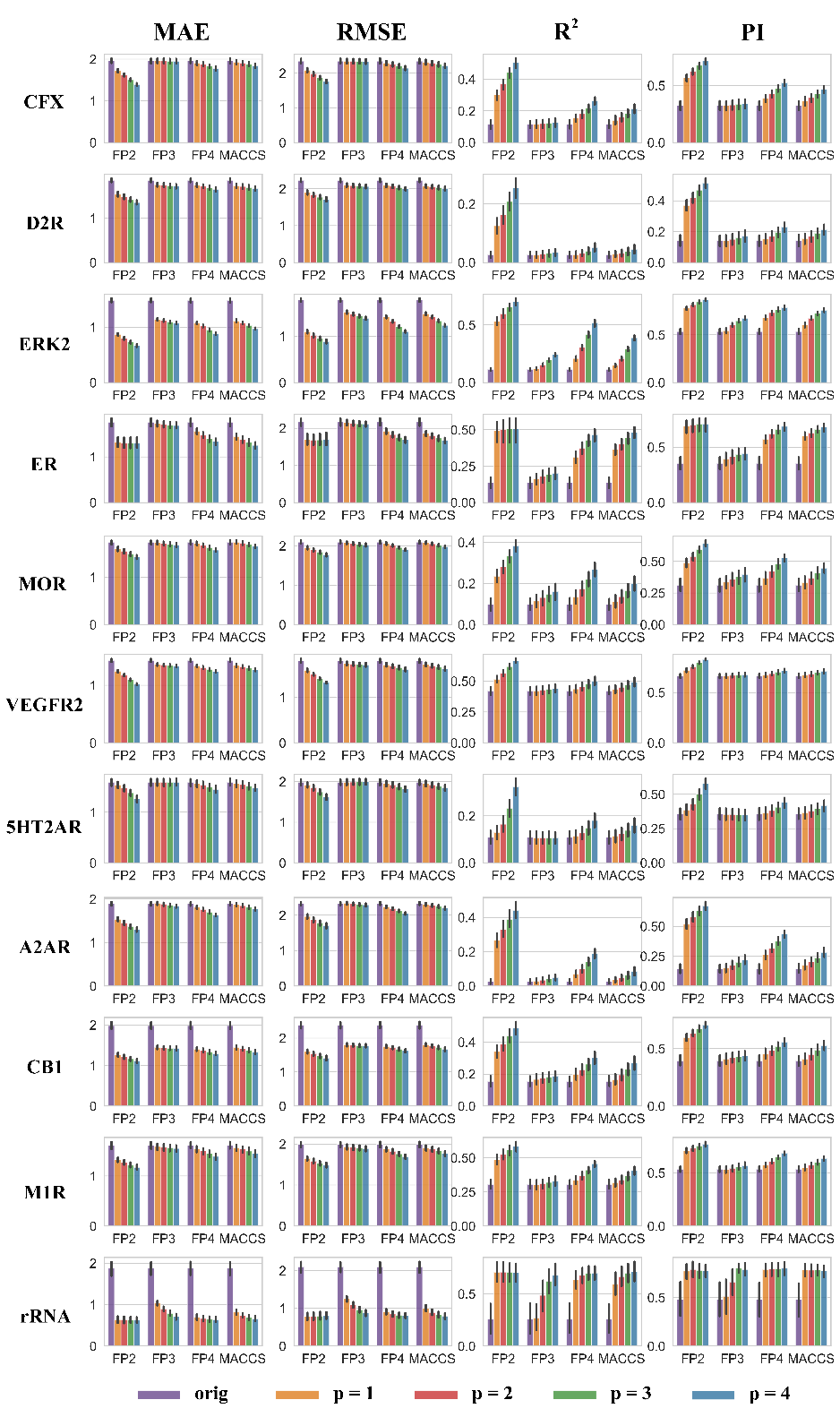
**

**Figure S1.** The comparison of RMSE (kcal/mol), MAE (kcal/mol), R^2^ and PI values before and after the calibration of the AutoDock vina docking scores for 11 drug receptors under the conditions of different fingerprint type and chemical structure effective function, *S^p^*, where *p* takes a value of 1, 2, 3 or 4. “*orig*” refers to original docking scores before the calibration.

**
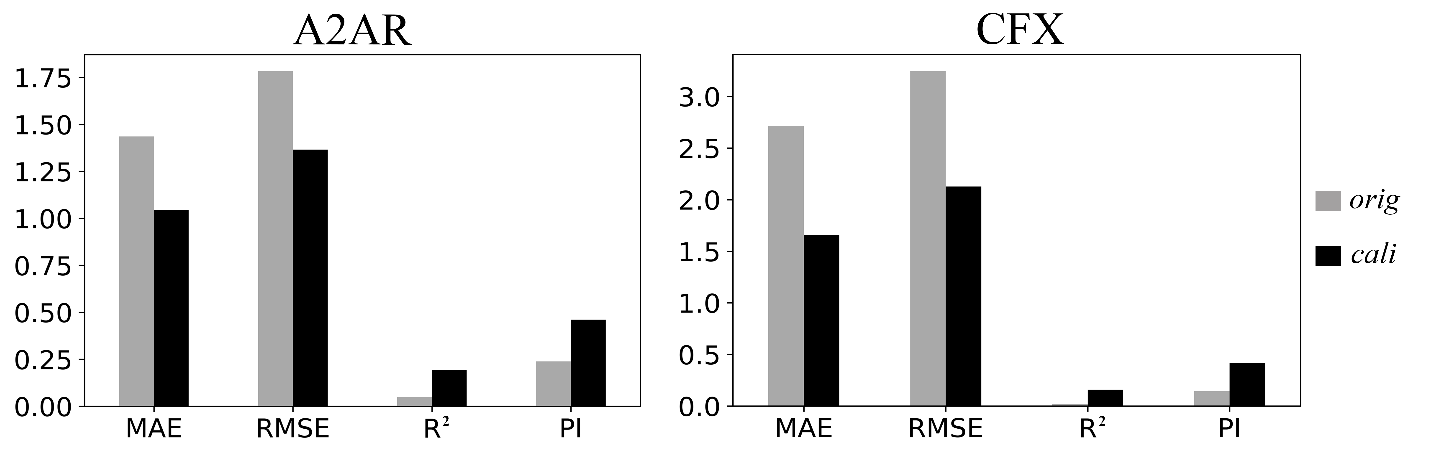
**

**Figure S2.** The comparison of RMSE (kcal/mol), MAE (kcal/mol), R^2^ and PI values before and after the calibration of the Glide docking scores for A2AR and CFX external test sets. “*orig*” refers to original docking scores before the calibration and “*cali*” refers to the docking scores after the calibration.


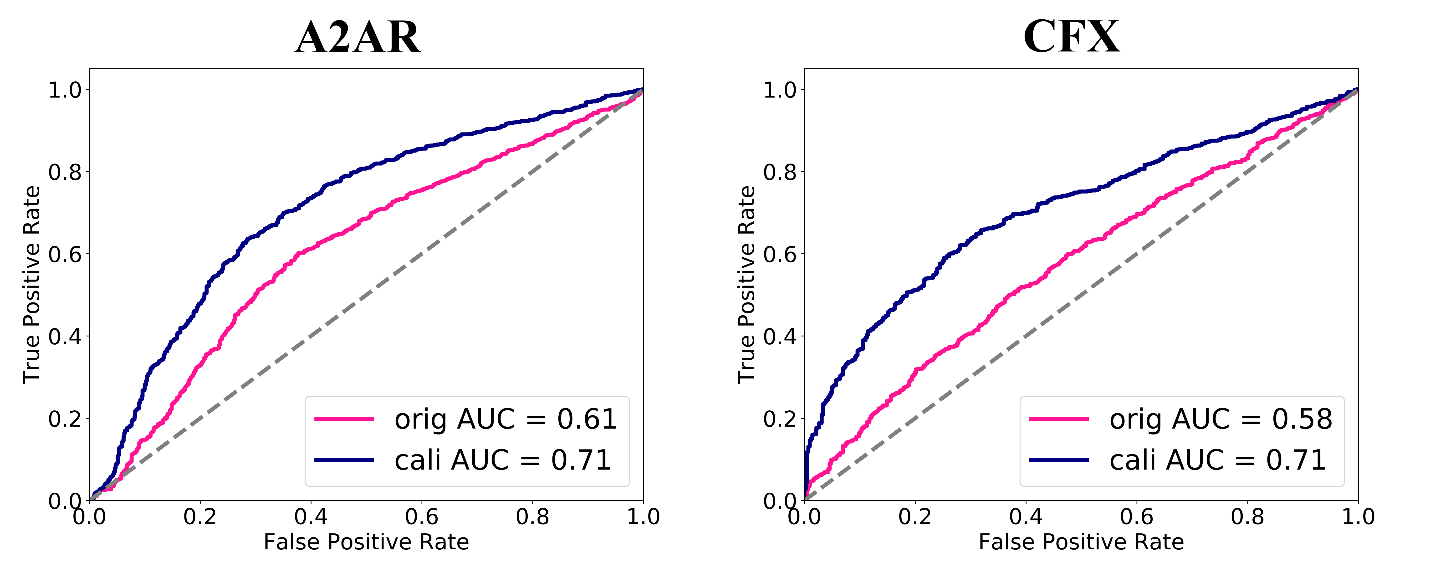


**Figure S3**. The ROC curves of screening results before and after calibration of the Glide docking scores using the best hybrid scoring function (FP2 fingerprint with CSE=S4) for A2AR and CFX external test sets. “*cali*” and “*orig*” represent the calibrated and original docking scores, respectively.
